# The patent buyout price for human papilloma virus (HPV) vaccine and the ratio of R&D costs to the patent value

**DOI:** 10.1371/journal.pone.0244722

**Published:** 2021-01-11

**Authors:** Mario Songane, Volker Grossmann

**Affiliations:** 1 Department of Economics, University of Fribourg, Fribourg, Switzerland; 2 Institute for the Study of Labor (IZA), Bonn, Germany; 3 CESifo, Munich, Germany; Rudjer Boskovic Institute, CROATIA

## Abstract

Human papillomavirus (HPV) is responsible for almost all of the 570,000 new cases of cervical cancer and approximately 311,000 deaths per year. HPV vaccination is an integral component of the World Health Organization’s (WHO) global strategy to fight the disease. However, high vaccine prices enforced through patent protection are limiting vaccine expansion, particularly in low- and middle-income countries. By limiting market power, patent buyouts could reduce vaccine prices and raise HPV vaccination rates while keeping innovation incentives. We estimate the global patent buyout price as the present discounted value (PDV) of the future profit stream over the remaining patent length for Merck’s HPV vaccines (Gardasil-4 and 9), which hold 87% of the global HPV vaccine market, in the range of US$ 15.6–27.7 billion (in 2018 US$). The estimated PDV of the profit stream since market introduction amounts to US$ 17.8–42.8 billion and the estimated R&D cost to US$ 1.05–1.21 billion. Thus, we arrive at a ratio of R&D costs to the patent value of the order of 2.5–6.8%. We relate this figure to typical estimates of the probability of success (POS) for clinical trials of vaccines to discuss if patent protection provides Merck with extraordinarily strong price setting power.

## 1. Introduction

Cervical cancer, a disease mainly caused by human papillomavirus (HPV), is the fourth most common type of cancer in women with approximately 570,000 new cases and 311,000 deaths per year globally [1–3]. By 2030 cervical cancer is projected to cause around 474,000 deaths in women annually. In addition, HPV induced anogenital cancers and genital warts in males are major causes of morbidity and represent a significant health burden. Furthermore, HPV infection has been associated with cancers of the anus, vulva, vagina and penis [4, 5].

The vast majority (95%) of deaths caused by cervical cancer and around half of the world’s new cases are in low-income countries (LIC) and middle-income countries (MIC) [6–9]. The highest age-standardized incidence rate is observed in countries in South Asia and Sub-Saharan region, where the vast majority of LIC and MIC are located [10]. The high prevalence of cervical cancer in these countries is caused by low coverage of HPV vaccination. In 2017 only 6% of LIC and 8% of lower-middle income countries introduced the new and effective HPV vaccines [11].

High vaccine prices, enabled by patent protection, is considered one of the main factors limiting the expansion of HPV vaccination in developing countries [12, 13]. Pharmaceutical companies that develop and manufacture vaccines frequently face demands to lower vaccine prices in order to make them affordable to poorer countries. The typical counterargument is that lower prices could induce companies to withdraw certain vaccines from the market or reduce research and development (R&D) investments for new vaccines [14, 15].

There are two strategies to promote vaccine R&D: pull programmes that provide financial reward to companies that develop successful vaccines and push programmes that provide direct funds for research. Pull programmes include prizes, compulsory licensing and patent buyouts whereas push programmes include research grants and tax credit [16]. For lowering prices of pharmaceuticals and extending access, pull programmes that limit market power of pharmaceutical companies entail larger potential.

This study focusses on patent buyouts by government agencies to supply medicines at lower costs and make them affordable to poorer countries via licensing production to many competitors [17]. The literature has suggested to determine the patent buyout value by a second-price auction [17] or to design the patent buyout by manipulating prices to infer the demand function for a pharmaceutical [18]. Based on available data and forecasts, we estimate the patent buyout price as the present discounted value (PDV) of incumbent’s expected future profits over the remaining patent length for two HPV vaccines, Gardasil-4 and Gardasil-9, from 2020 onwards. We use the current and past pricing strategies in different markets by patent holder Merck jointly with sales data including future predictions provided by GAVI [19]. Our methodology requires to explicitly distinguish variable costs and fixed costs, and to account for marketing expenditure. Data on variable costs for materials, labor and capital are adapted from Clendinen *et al*. [12]. Our measure for fixed costs include overhead costs derived from data provided by Clendinen *et al*. [12] and marketing expenditure based on acquired data from ‘The Nielsen Company’. We come up with regional estimates for operating profits and a global patent buyout price. Although patent buyouts for LIC and MIC would be most desirable and could in principle be relatively cheap in view of the low revenues in these countries, focussing the patent buyout on these groups of countries may be infeasible. First, pharmaceutical companies may be reluctant to this solution because they fear parallel imports or a black market for generic drugs. They may also fear public debate on prices in high-income countries (HIC) or reference pricing in health systems based on the global market, once local supply by generic manufacturers at low prices make high price-cost margins in HIC transparent [20].

We also estimate the global value of the Gardasil patents at market entry, using a similar methodology as for deriving the patent buyout price, and derive estimates of R&D costs by identifying each clinical trial sponsored by Merck on www.clinicaltrial.gov and a literature search. We calculate the R&D costs based on previously estimated costs per subject [15], clinical trial site, and study [21]. Vaccine and drug R&D is divided in two stages, pre-clinical (*in vitro* and *in vivo* studies) and clinical trials (phase I-III). Most of the pre-clinical development of Gardasil-4 was performed by the National Cancer Institute (NCI), Georgetown University and University of Queensland who were the first to develop virus-like particle (VLP) technology used in the vaccine in the early 1990s [22]. Merck later acquired the licenses and took the then vaccine candidate to clinical testing [22].

We compute the ratio of estimated R&D costs for clinical trials to the patent value (PDV of the stream of profits from market entry to patent expiry). In theory, this ratio is the probability of success (POS) in finding an innovation that is required to just cover the R&D costs in expected value [23]. We thus compare our estimated ratio of R&D costs to the value of the Gardasil patents with POS estimates in the literature of moving vaccines for infectious diseases successfully from Phase I to approval. This strategy allows us to discuss whether the patent system gives vaccine producers more market power than needed to serve the goal of providing incentives to start clinical trials for a particular vaccine candidate.

Our estimated global patent buyout in year 2020 for Gardasil vaccines is in the range of US$ 15.6–27.7 billion (in 2018 US$), depending on assumed real annual return to investment in alternative uses, price estimates, and cost estimates. The patent value, we estimate between US$ 17.8–42.8 billion. These high values reflect our findings of high price-cost margins which could be around 100 for the U.S. and China and still well above 10 in MIC. The estimated R&D costs for both Gardasil vaccines combined lie in the range between US$ 1.05–1.21 billion. These figures imply a ratio of R&D costs and the patent value between 2.5–6.8%. We will argue that this figure is considerably lower than we would expect from the estimated POS for vaccines from Phase I to approval in the literature, suggesting that the patent holder earns extraordinarily high profits.

Estimates of R&D costs from confidential sources such as self-reported R&D costs from pharmaceutical companies and estimates of industry experts are impossible to assess for accuracy, representativeness, or sensitivity to outliers [15]. Our estimated R&D costs may be compared to two recent studies that also assessed the costs of R&D of new medicines based on publicly available data. Prasad and Mailankody [24] found that the median R&D costs for the 10 new molecular entities approved by the U.S. Food and Drug Administration (FDA) for oncologic indications, filed in the period 2006–2015 by firms that had no other drug in the U.S. market, was US$ 683.6 million. For novel (rather than next-in-class) drugs it was US$ 899.2 million, ranging from US$ 328.1 million to US$ 1950.8 million. Wouters *et al*. [25] estimated the R&D cost for new drugs approved by the FDA in the period 2014–2018 with publicly available data. Dividing the costs of clinical trials by phase-specific POS estimates to account for failed trials (which means that their estimates are not directly comparable to ours) they came up with median R&D costs of US$ 985.3 million.

### 1.1 Background: Vaccines for HPV

HPV is a group that encompasses over 100 viruses and 15 of them have been shown to be responsible for almost all cases of cervical cancer: HPV 16, 18, 31, 33, 35, 39, 45, 51, 52, 56, 58, 59, 68, 73, and 82 [26]. One of the challenges to create a vaccine and treatment for HPV is the fact that an individual can be infected by more than one HPV type [27]. Globally, HPV 16 and 18 are responsible for approximately 70% of all cases of cervical cancer. HPV type 16 causes mainly squamous cell carcinoma whereas HPV 18 causes the less aggressive adenocarcinoma [28]. In Africa, HPV 16 and 18 are responsible for 43.7–90.2% of the invasive cervical cancer cases [29].

The two most widely used prophylactic vaccines in the market are Gardasil-4 (Merck, NJ, USA) and Cervarix (GlaxoSmithKline-GSK, Middlesex, UK). Gardasil-4 was approved for both U.S. and European markets in September 2006 whereas Cervarix was approved in September 2007 [30, 31]. They target HPV L1 major capsid protein that assemble to form VLPs with a morphology similar to the HPV native virions and generate robust antibody responses against the targeted HPV types [32]. Both vaccines contain VLPs for HPV 16 and 18, which cause cervical cancer, but Gardasil-4 also has VLPs for HPV 6 and 11, which cause benign genital warts. The U.S. Food and Drug Administration (FDA) approved Gardasil-4 for immunization against HPV in males and females aged between 9–26 years whereas Cervarix is approved only for females aged between 10–25 years [32]. Merck introduced a new HPV vaccine, Gardasil-9, in the U.S. and Europe in 2014 and 2015 respectively. In addition to the four HPV types covered in Gardasil-4, the new vaccine also provides protection against HPV types 31, 33, 45, 52 and 58 [33].

Vaccination is an integral part of the WHO global strategy to combat HPV infections and related diseases [9]. According to WHO recommendations, girls aged 9–14 years should receive two doses of Gardasil-4 whereas older age groups receive three doses [9]. When given at recommended doses, Gardasil-4 has been shown to induce antibody protection against HPV types covered by the vaccine for at least 5 years [27, 34]. In males aged 16–26 years, Gardasil-4 has been shown to provide 90.4% efficacy against lesions related to HPV types covered by the vaccine [35].

It is estimated that 70–80% of females in pre-pubertal age have to be vaccinated in order to achieve herd immunity [36]. Although HPV vaccination has been introduced in 81 countries, the high costs of the vaccine and the fact that it requires the delivery of two or three doses over a period of 6 months makes it a significant financial and structural burden to most countries in the world. For this reason, most LIC and MIC struggle to maintain HPV vaccination without the aid of other countries and global organizations such as the WHO, the Global Vaccine Alliance (GAVI), United Nations International Children's Emergency Fund (UNICEF) and Pan American Health Organization (PAHO) [31]. In these countries, it has been estimated that HPV vaccination has the potential to reduce the lifetime risk of cervical cancer by 31–60% [37].

Academic institutes in the U.S. and Australia first developed the technology employed in the VLP-based vaccines in the early 1990s. Merck and GSK then improved on the original invention and performed the subsequent steps required to bring the vaccine to the market [22]. Merck’s Gardasil-4, the first VLP based vaccine, was patented in the U.S. in 1998 and introduced in the market in 2006 [38]. Between the first patent approval and 2010, 81 HPV vaccine related patents were granted in the U.S. with Merck leading the way with 24 granted patents [22]. Table 1 shows global revenues of Gardasil and Cervarix between 2006 and 2018, suggesting that Gardasil has dominated with an average market share of 87% in the period 2007–2018. For this reason and because it is effective against more HPV types, we focus on Gardasil.

**Table 1:** Global revenue of Gardasil and Cervarix from 2006–2018.

	Gardasil (in million US$)*	Cervarix (in million US$)**
**2006**	234.8	0
**2007**	1,480.6	20.1
**2008**	1,402.8	248.75
**2009**	1,118.4	306.68
**2010**	988	367.84
**2011**	1,209	814.66
**2012**	1,631	423.9
**2013**	1,831	261.44
**2014**	1,738	202.96
**2015**	1,908	137.28
**2016**	2,173	106.92
**2017**	2,308	172.86
**2018**	3,151	180.78

* Includes sales of Gardasil-4 and Gardasil-9. From 2016 onwards, only Gardasil-9 was sold in the U.S. [39, 40].

** Original revenues are in Great Britain Pound (£). We used the exchange rate of the respective year on July 2 [41] to calculate the revenue in US$.

*Sources*: Merck [33, 42–46], GSK [47–52].

There is no centralized database to obtain information about the status of the various patents related to a particular vaccine in different countries/regions. In the U.S., Gardasil patents expire in 2028, and similarly in other advanced countries [33, 53]. We focus on patent buyout prices for all country groups and total patent values until 2028.

The biggest factor that will drive the increase in global demand of HPV vaccine is the introduction of HPV vaccination in China, India and Indonesia (three of the four most populous countries in the world). These countries are expected to represent approximately 1/3 of the global market by 2030 [19, 54]. China, India and Indonesia delayed the introduction of Gardasil-4 and Gardasil-9 in their public health programmes due to concerns over safety, effectiveness of the vaccine across different age groups and price. China approved the introduction of Gardasil-4 and Gardasil-9 in 2017 and 2018 respectively, Indonesia introduced Gardasil-4 in late 2015 and India did so in 2018 [55–58].

### 1.2 The benefit of HPV vaccine patent buyout

Our focus on HPV vaccination derives, apart from data availability issues, from its importance for global health and the prevalence of HPV related diseases particularly in developing countries. Patents like those for HPV vaccines provide intellectual property protection that bars entry of generic competitors for an extended period of time, therefore awarding monopoly price setting power. Because inventions use prior knowledge that is protected by patents, it is *a priori* not evident that patents are stimulating innovation [59]. One of the strategies to counter patents is to challenge them on court. However, it is a long and expensive process that most small and medium sized companies cannot afford. Another strategy is to work around patents. This, however, is very challenging because patents often cover various stages of the vaccine development and manufacturing process [60]. Working around patents increases uncertainty and costs, thus constituting a barrier for vaccine development.

Under the agreement on trade-related aspects of intellectual property rights (TRIPS), administered by the World Trade Organization (WTO) and enforced in 1995, member countries with an industry capable of manufacturing vaccines must enforce patent protection of medicines and biological products [60]. There is evidence that TRIPS limited access to pharmaceuticals formerly manufactured by local suppliers [20]. However, under the agreement, the least-developed countries [61] are not obliged to provide patent protection in general until 2021, and on medicines (including vaccines) specifically until 2033 [62].

Brazil, India and China have a large generic pharmaceutical industry supplying 64% of vaccines purchased by UNICEF and 43% of vaccines procured by GAVI [22]. In addition to manufacturing generic vaccines, these countries are also capable of developing HPV vaccines themselves [22]. A common strategy used in the pharmaceutical industry to limit competition from Brazil, India and China is to apply for patents in these countries. There has been over 100 HPV vaccine related patent applications with GSK and Merck, the two companies that dominate the HPV vaccine market, having by far the highest number of patent applications [60].

WHO has launched a global effort to eliminate cervical cancer by promoting introduction of HPV vaccination in all countries [54]. Accordingly, 48 GAVI-supported countries announced plans to introduce multi-age cohort HPV vaccination and protect approximately 40 million girls by 2020 [54]. However current manufacturers could not match the demand leading to supply shortages and the goal been reduced to protect only 14 million girls [63]. Moreover, the introduction of HPV vaccination in China, India and Indonesia adds significantly to global demand. From 2023 the demand for HPV vaccines is likely to exceed production capacity [19].

Another factor limiting the expansion of HPV vaccination are the high prices of HPV vaccines that are prohibitive to GAVI and PAHO supported LIC and MIC [13]. To date, 74% of the 81 countries that have introduced the HPV vaccination self-procure the vaccines [54].

The discussion strongly suggests that the entrance of new manufacturers is crucial to ensure sufficient supply and lower HPV vaccine prices, thus increasing static economic efficiency by mitigating price setting power of the incumbent [17]. Given the currently high price-cost margins we display in this research, patent buyout for the market leader Gardasil-4 and Gardasil-9 that puts the right to manufacture and sell the vaccine into public domain could allow fast entry of competitors and development of new and less expensive technology (i.e. plant-based production). Importantly, a patent buyout price equal to the estimated PDV of the expected stream of future profits would most likely fully compensate the innovator for the expected loss in profits from market transactions while keeping incentives for future innovators unchanged (i.e. not compromising dynamic efficiency).

Despite these arguments in favor of the patent buyout at the suggested price, we do not aim to calculate the net social benefit from the patent buyout because of several difficulties to predict the entry response of generic manufacturers and the price responses by the patent holder. First, for some period of time after the buyout, Merck would likely continue to be the major supplier and prices may remain significantly above marginal costs. It is also plausible that some LIC and MIC countries would choose to postpone introducing HPV vaccination (or postpone scaling up pilot programs) until after generics enter the market, if they knew it was imminent. Likewise, GAVI and the PAHO revolving fund would aim at negotiating lower prices.

## 2. Methodology and data

We first propose our methodology to estimate the patent buyout price and to assess the extent of price setting power in the market of HPV vaccination. We then discuss the data and assumptions for our estimations.

### 2.1 Theoretical considerations on the patent buyout price

Our first goal is to estimate the PDV of the expected future stream of real profits for the vaccine Gardasil, starting from year *T*, i.e. year 2020 this gives us the patent buyout price (with caveats discussed in Section 4) for the vaccine Gardasil under the assumption of risk-neutrality. Arguably, large pharmaceutical companies like Merck can be considered risk-neutral as they are owned by shareholders that can hold diversified asset portfolios. Moreover, they have diversified product portfolios and can diversify R&D effort. By contrast, a (small) risk-averse company facing market uncertainty would agree to a patent buyout price that is lower than the PDV of expected future profits.

To calculate the time path of profits, we need estimates for sales prices, variable costs, and fixed costs. We assume that the variable cost per unit (dose) is independent of the number of produced doses such that total variable costs are proportional to the units sold. They consist of:

- user costs of physical capital (equipment, building, pipes),
- costs for materials needed to express and purify the VLPs of the targeted HPV types,
- costs for filling and packaging (staff and material),
- costs for operating labor (manufacturing operators, quality assurance, and quality control operators).

Fixed costs consist of:

- overhead labor costs for managing and supervising the manufacturing process in the facilities used for producing the vaccine,
- labor costs for maintaining the facilities and equipment,
- marketing expenditure (direct-to-consumer advertising and promotion on health providers).

We follow Clendinen *et al*. [12] to treat maintenance costs as fixed costs, albeit we checked that our results are not significantly affected by this choice. We depart from Clendinen *et al*. [12] by modelling capital investments as variable rather than fixed costs to account for the possibility of alternative uses (“user costs of capital”), as is standard in economics.

Legal costs for patent defence, patent infringement disputes, product liability litigations, commercial litigations, government proceedings or environmental matters should generally be considered as fixed costs. Merck gives a detailed account of these costs in its annual reports. However, so far, no legal proceedings or costs were reported for Gardasil-4 and Gardasil-9. Also notably, R&D expenditures are incurred *ex ante* and thus cannot be considered being part of the fixed cost [64].

That said, we still miss some labor-intensive fixed cost components that are private information like building relations with suppliers of material, training costs and costs for developing the manufacturing to scale process and packaging design. There is reason to assume, however, that in the present context these costs are small. First, large scale manufacturing of VLP based vaccines was already being done in the 1990s and early 2000s for a variety of VLPs vaccines against HEV, influenza, BTV, rotavirus and PPV [65]. All the techniques, technologies and equipment were routinely used at the time when Gardasil was being developed. Moreover, for manufacturing its other vaccines Merck had already established supplier relations for the bulk of the material components to produce Gardasil.

We also miss distribution costs. However, the vast majority of distribution costs are covered by government and NGO’s. In the U.S., the Centers for Disease Control (CDC) Vaccine Supply and Assurance Branch (VSAB) oversees all aspects of its vaccine purchase and distribution in the country [66]. In the EU, the most common strategy is for the manufacturers shipping vaccines to distribution centres and wholesalers, who are then responsible for the distribution within the country [67]. In GAVI supported countries the distribution is carried by governments and the various partnering NGOs, i.e. WHO, UNICEF, etc. [67].

We index the country (group) in which Gardasil is sold by *j*∈{1,2,…,*J*} and denote the time horizon by *T* (U.S. patent expiry year 2028 in our application). We use information about the time paths of the predicted future number of doses sold at the regional level, ${\left\{y_{jt}\right\}}_{t=\underline{T}}^{T}$. We deflate all prices and costs to its 2018 US$ value for obtaining the time series of real profits. For data availability reasons, we assume that the variable unit cost is time-invariant in *real* terms. In year *t*, operating profits from sales to country group *j* are then given by

$$\pi_{jt}=(p_{jt}-c)y_{jt},$$ (1)

where *p*_*jt*_ and *y*_*jt*_ denote sales prices and number of doses in region *j* and year *t*, respectively, and *c* the variable costs per dose.

Based on Eq No. 1, we can then estimate the present discounted value (PDV) of the stream of operating profits from period *T* onwards in country group *j* until time *T* as

$$\Pi_{j}(\underline{T})≔\sum_{t=\underline{T}}^{T}\frac{\pi_{jt}}{{\left(1+r\right)}^{t-\underline{T}}}$$ (2)

where *r* is the real annual return to investment in alternative uses (like deposits in banks, equity holding, government bonds, etc.). We present our estimates for the annual real interest rate *r* in the range of 3–7%.

Fixed costs are typically not region-specific, as production takes place in few facilities and the product is shipped from there. We denote real global fixed costs in year *t* by *f*_*t*_ and the PDV of the fixed cost stream from period *T* until time *T* by

$$F(\underline{T})≔\sum_{t=\underline{T}}^{T}\frac{f_{t}}{{\left(1+r\right)}^{t-\underline{T}}}.$$ (3)

Thus, the global patent buyout price in year *T* for the considered regions is given by

$$V(\underline{T})≔\sum_{j=1}^{J}\Pi_{j}(\underline{T})-F(\underline{T}).$$ (4)

We assume that both variable unit cost *c* and fixed costs *F* are the same for Gardasil-4 and Gardasil-9.

Arguably, buying out a patent in *T* at the PDV of future profits, *V*(*T*), fully compensates the patent holder for its profit losses associated with lower price setting power and is thus incentive-compatible.

### 2.2 Required probability of success (POS) to break even

We also aim to estimate the patent value from the perspective of the date introduced in the market and compare it with R&D costs. Gardasil-4 was introduced in September 2006, i.e. 2007 was the first full year Gardasil was sold. To generate conservative estimates of the patent value and since we do not have price information for 2006, we neglect profits for 2006 and include marketing costs for 2006 in total fixed costs for 2007.

Denoting the first full year of market introduction *s*≤*T*, the total patent value is given by *V*(*s*). If the market for innovations is characterized by free entry and firms are risk-neutral, the expected value of an innovation (accounting for a potentially high risk of R&D failure) equals R&D costs [23]. Formally, let *μ* denote the POS in the innovation process, such that *μ∙V*(*s*) is the expected patent value (in case of R&D failure, with probability 1−*μ*, profits are zero). A firm is incentivized to conduct the innovation project as long as *μ∙V*(*s*)≥*R*&*Dcosts*. The POS required to break even in expected value is given when the inequality is binding. It is defined as

$$\mu_{0}≔\frac{R\&D\;costs}{V(s)}$$ (5)

Our data allows us to estimate the right-hand side of Eq No. 5. To assess market power, we compare the estimated *μ*_0_ with typical estimates of the average POS for clinical trials of other vaccines reported in the literature. Higher price setting power would increase *V*(*s*) and thus decrease *μ*_0_. A value of *μ*_0_ that is considerably lower than the average POS found for other vaccines could therefore indicate excessive price setting power of the patent holder.

In our context of HPV vaccines, the POS is the cumulative probability of moving from the three phases of clinical trials to approval. Recall that Gardasil-9 was an improvement of Gardasil-4, the first VLP-based HPV vaccine approved by the FDA [68]. Gardasil-4 and Gardasil-9 are typically not sold in parallel within the same region. Thus, for obtaining *μ*_0_, we sum up both the PDV of profits from both Gardasil-4 and Gardasil-9 since market introduction until patent expiry when computing *V*(*s*), the denominator of Eq No. 5, and sum up the R&D costs for both to compute the numerator.

### 2.3 Data and assumptions

We now outline the data sources, how we extrapolate missing information, and how we deal with measurement problems and uncertainty with respect to future values. We convert all monetary variables to 2018 US$, using the U.S. consumer price index [69]. Detailed statistics and derivations based on the assumptions to extrapolate missing data are relegated to the “S1 File” on the article.

#### 2.3.1 Data for operating profits

According to Eq No. 1, calculating regional operating profits requires estimates of regional sales prices (*p*_*jt*_), regional number of doses sold (*y*_*jt*_), and variable unit cost (*c*). The prices of HPV vaccines vary greatly depending on procurement agreement and countries’ income [9]. Merck and GSK have agreements with organizations such as GAVI, that mediates purchase of vaccine to developing countries in Africa and Asia and PAHO, that mediates vaccine purchase to LIC and MIC in South and Central America [70, 71]. When Gardasil-4 was introduced in the U.S. market, the median price was US$ 96.75 per dose to CDC funded programs and US$ 120.5 per dose to the private sector. It was the most expensive vaccine in the world at the time [40, 72]. In 2018, Gardasil-9 was sold at a price of US$144.2 and US$ 217.1 to CDC and the private sector, respectively (S2 Table in S1 File) [40]. The median U.S. private sector price for Gardasil-9 in 2016 was about twice as high than in other HIC and about 25% higher than in China [73, 74]. The median Gardasil-4 price in 2016 was about US$ 20 in MIC [9], US$ 4.50 for sales mediated by GAVI [9] and in-between in India [75] and Indonesia [76] (S1 Table in S1 File). We assume that from 2019 onwards regional prices stay the same as the latest one observed (in 2018 US$), taking Gardasil-9 prices for HIC and China and Gardasil-4 prices for the other regions. Due to the price difference between the U.S. and other HIC, we treat the U.S. as separate region, assuming that half of the units sold in the U.S. are mediated by CDC funded programs and the other half by the private sector.

Through its own databases and interviews with stakeholders (i.e. national vaccination programme managers, industry leaders and experts), GAVI [19] estimated the number of HPV doses sold between 2010–2017 by country group and forecasted quantities for 2018–2027, however, without distinguishing the U.S. and other HIC. To break down the number of units sold between the U.S. and other HIC, we use the global sales revenues in Table 1 and the revenue for the U.S. for 2015–2017 that is additionally available in Merck’s annual report [77] to find that about three quarters of the global sales revenue is earned in the U.S. (S3a Table in S1 File). Assuming that this has also been the case in 2007–2014, information on prices and revenues for the U.S. allow us to distil the number of doses (revenue divided by price) in the U.S. (S3b Table in S1 File). Moreover, although GAVI [19] did not estimate the number of doses sold from 2007–2009, we obtain the number of doses sold in other HIC from the additional information that in this time period Gardasil was sold in HIC only (S3c Table in S1 File). For the period 2018–2027, we assume that the share of the number of doses in the U.S. within HIC is the same as the average in 2007–2017 (about 60%) (S4 Table in S1 File). We suppose that the number of doses in patent expiry year 2028, for which we do not have data, is the same as predicted for 2027.

We account for measurement problems of past vaccine doses’ sales and uncertainty in the predictions for future sales by creating a “low” and “high” scenario by deducting and adding 20% of the sales of doses sold to the middle estimates that we derive by the outlined procedure (S5a and S5b Table in S1 File). For instance, the predictions are based on the current dosage recommendation for HPV vaccination. However, a recent clinical trial in India showed that a single dose of Gardasil induces a robust and sustained immune response against HPV 16 and 18 (although slightly inferior to those induced by the standard 3-doses vaccination) and the antibody levels were stable over a four-year period [78]. Interestingly, the cumulative incidence of HPV 16 and 18 infections was similarly low in those that received one, two or three doses of Gardasil [78]. If more studies show further evidence that a single dose is sufficient to protect against HPV-16 and 18 infection, then the forecasted number of doses required could be significantly lower. Likewise, it is possible that future demand is underestimated because of regional changes in health strategies.

Merck’s Gardasil-4 is produced from a combination of non-infectious and non-oncogenic VLPs that mimic the virion structure of the infectious particles of common HPV strains. It contains four VLPs of HPV type 6, 11, 16, and 18 each absorbed onto aluminium hydroxyphosphate sulphate (AAHS) adjuvant. The manufacturing process has four steps: cultivation, extraction, purification type-specific VLPs from L1-recombinant producer cells, filling and packaging [12]. Associated variable costs consist of annual costs for labor, per-batch costs for raw materials, and capital costs. We take over the “low” and “high” estimate of annualized capital costs (for building, equipment and pipes) from Clendinen *et al*. [12] (S6a Table in S1 File). Based on the average number of doses sold between 2010–2017, we obtain the unit variable capital costs of Gardasil-4 (S6b Table in S1 File). Similarly, we use the estimate of the unit costs for materials provided by Clendinen *et al*. [12]. The “high” estimate is based on the life science and technology companies list prices in 2013 (in 2018 US$) and the “low” estimate on a 40% discount on the cost for materials (S7 Table in S1 File). Regarding labor costs, we deviate from Clendinen *et al*. [12] by distinguishing variable and fixed labor costs, taking over their range of salary estimates and required staff for the different occupations. We treat salaries of manufacturing operators, quality assurance operators, quality control operators and fill/pack staff per unit as variable costs (S8 Table in S1 File).

#### 2.3.2 Data for fixed costs

Fixed manufacturing costs consist of management and supervisor overhead costs, and the costs of maintenance of the facility, and the equipment according to ‘Good Manufacturing Practices’ guidelines (electricity, heating, cooling and operation of the machinery) (S10a Table in S1 File). As such “factory and administrative overhead” costs are not readily available, we take over the assumption in Clendinen *et al*. [12] that they correspond to 45% of the total cost for other personnel and material (S10b Table in S1 File).

The second component of fixed costs are marketing expenditures. We obtained the time series of Merck’s direct-to-consumer advertising (DTCA) expenditure to promote Gardasil-4 and Gardasil-9 in the U.S. since market entry from ‘The Nielsen Company’. We use their additional information that DTCA spending in the U.S. is 90% of the global spending and create two scenarios for the missing marketing expenditure of health care providers (S11 Table in S1 File). First, for the low-cost scenario we assume that 2/3 of global marketing spending is on DTCA. The scenario is motivated by the evidence that Merck’s marketing strategy focused to a large extent on raising awareness of the general public of the risk of cervical cancer. Gardasil was marketed as vaccine against such cancer, aiming to increase demand from consumers for HPV vaccination [79]. Second, for the high-cost scenario, we assume that global marketing spending on health care providers is twice as high than marketing spending on DTCA. The range for the marketing cost split is consistent with evidence on other pharmaceuticals [80]. Our data for DTCA spending suggests that marketing expenditures decline quickly over the life-cycle of a new vaccine. We assume that annual spending from year 2020 onwards equals, in real terms, the average annual spending in the last three available years (2017–2019).

#### 2.3.3 Data for R&D investment

The elements discussed so far allow us to calculate the total patent value, *V*(*s*), according to Eqs No. 1–4. To apply Eq No. 5, we relate these to R&D costs. We add the costs of clinical trials (phase I to III). Notably, we do not include the costs of clinical development because it was mainly performed by academic institutions [22]. To estimate the costs of each clinical trial we performed a literature review coupled with a search in www.clinicaltrial.gov to identify the Gardasil related clinical trials sponsored by Merck in each phase. To calculate the costs on subjects (set-up, recruitment, administration and support) for each clinical phase we use the phase-specific estimates of costs per subject in Light *et al*. [15] (S12 Table in S1 File) and multiply them with the number of subjects identified in www.clinicaltrial.gov. In addition to subject-related costs, there are site costs (for recruitment and retention of subjects as well as administrative and site monitoring) and study costs (data collection and management, institutional review board approvals and amendments, source data verification, overheads and other costs) (S13 Table in S1 File). Both site and study costs per trial in the three phases are taken from Sertkaya *et al*. [21] and multiplied with the number of clinical trials in each phase for Gardasil-4 (S14a Table in S1 File) and Gardasil-9 (S14b Table in S1 File).

According to www.stats.oecd.org, capital R&D costs in business enterprises for “basic pharmaceutical products and pharmaceutical preparations” are typically around 10% of non-capital R&D costs (e.g. 10.4% in the UK 2016, 10.1% in Germany 2017, 11.3% in Japan 2018; retrieved July 19, 2020). We add 10–15% to the subject-related, site and study costs to account for R&D capital costs.

## 3. Results and discussion

### 3.1 Operating profits

Based on the assumptions and data presented in Section 2.3, Table 2 displays the middle estimates for number of doses sold in different (groups) countries between 2007–2017 and forecasted until 2027. The forecasts in Table 2 suggest that annual future sales are slightly increasing in HIC and MIC. Globally, they more than triple because of sales increases in emerging markets and those mediated by GAVI.

**Table 2:** Number of Gardasil doses sold in different countries and country groups between 2010–2017 and forecasted quantities until 2027 (middle estimates), in millions.

**Year**	**HIC total**	**U.S.**	**Other HIC**	**MIC**	**GAVI**	**India/ Indonesia**	**China**	**Total**
2007	19.60	10.42	9.18	0.00	0.00	0.00	0.00	19.60
2008	17.88	9.50	8.38	0.00	0.00	0.00	0.00	17.88
2009	13.95	7.25	6.70	0.00	0.00	0.00	0.00	13.95
2010	15.66	6.32	9.34	6.09	0.00	0.00	0.00	21.75
2011	20.88	8.18	12.70	11.31	0.00	0.00	0.00	32.19
2012	18.27	10.66	7.61	13.92	0.00	0.00	0.00	32.19
2013	17.40	11.54	5.86	13.92	0.87	0.00	0.00	32.19
2014	15.23	10.13	5.10	11.31	0.87	0.00	0.00	27.41
2015	14.79	10.81	3.98	9.57	1.74	0.00	0.00	26.10
2016	15.66	11.39	4.27	9.57	2.61	0.87	0.00	28.71
2017	15.66	9.75	5.91	9.57	1.74	0.87	0.00	27.84
**Subtotal (2007–2017)**	**133.55**	**78.77**	**54.77**	**85.26**	**7.83**	**1.74**	**0.00**	**228.38**
2018*	14.79	8.84	5.95	10.44	5.22	0.87	0.00	31.32
2019*	15.23	9.10	6.12	12.18	23.49	0.87	0.00	51.77
2020*	16.53	9.88	6.65	13.05	34.80	0.87	0.00	65.25
2021*	15.66	9.36	6.30	13.05	26.97	13.92	0.00	69.60
2022*	14.79	8.84	5.95	13.05	28.71	25.23	0.87	82.65
2023*	13.92	8.32	5.60	13.05	31.32	23.49	2.61	84.39
2024*	13.05	7.80	5.25	13.92	21.75	24.36	3.48	76.56
2025*	12.18	7.28	4.90	14.79	27.84	21.75	3.48	80.04
2026*	12.18	7.28	4.90	14.79	26.10	20.01	4.35	77.43
2027*	12.18	7.28	4.90	14.79	25.23	18.27	6.09	76.56
**Subtotal (2018–2027)**	**140.51**	**84.02**	**56.48**	**133.11**	**251.43**	**149.64**	**20.88**	**695.57**

*Source*: Own calculations displayed in S1-S4 Tables in S1 File.

Table 3 displays the range of variable costs per dose (1.00–1.59 US$) and its composition. We see that costs for materials, filling and packaging are responsible for about one third of variable costs. Filling and packaging represents almost 60% of these costs, being composed of the wholesale cost of the vial, cap and stopper (for single-dose packaging) at US$ 0.21 per dose plus secondary packaging materials at US$ 0.10 per dose [12]. About one quarter of variable costs are labor earnings. The remaining costs are capital costs (about half for building and half for equipment).

**Table 3:** Variable costs for manufacturing Gardasil, in 2018 US$.

	Low estimate	High estimate
Costs for materials, filling and packaging per million doses	323,947.53	539,912.55
Variable labor costs per million doses	243,506.49	340,909.09
Variable capital costs per million doses	428,067.87	711,251.24
**Total variable costs per million doses**	995,521.89	1,592,072.88
**Total variable costs per dose**	1.00	1.59

*Source*: Own calculations displayed in S6b-S8 Tables in S1 File.

It is interesting to look at price-cost margins. Even based on the high estimate for variable costs, the mark-up factor (price divided by unit variable costs) in 2016 is around 100 for the U.S. and China, twelve in MIC and almost three for sales mediated by GAVI.

We derive two estimates for regional operating profits. The “high” estimate combines the 20% upward deviation from the middle estimates of the number of doses in Table 2 with the “low” unit cost estimate in Table 3 (regional prices are given in S1 and S2 Tables in S1 File), applying Eq No. 1, and vice versa for the “low” estimate. Based on those, we apply Eq No. 2 to derive the PDV ranges of the future (2020–2028) and total (2007–2028) stream of operating profits. Table 4 presents the results for three interest rates, *r* = 0.03, *r* = 0.05 and *r* = 0.07. We estimate the PDV of future operating profits from the perspective of year 2020 in the range of US$ 16.45–28.94 billion, and the total PDV of the stream operating profits from the perspective of year 2007 in the range of US$ 19.82–44.25 billion. Reflecting the comparatively high sales prices in the U.S., half of the future profits are earned in the U.S. The profit share earned in the U.S. is even higher when considering the time period 2007–2028, as the sales share in the U.S. market was higher in the past than predicted for the future, reflecting market expansion to less developed countries.

**Table 4:** Estimated PDV of the stream of operating profits (in million 2018 US$) for the periods 2020–2028 and 2007–2028, applying Eq No. 2.

	Interest rate (*r*)	Estimate	2020–2028	2007–2028
**U.S.**	**0.03**	**High**	14,210.23	27,584.81
		**Low**	9,442.37	18,319.64
	**0.05**	**High**	13,297.34	23,009.99
		**Low**	8,835.78	15,280.53
	**0.07**	**High**	12,488.32	19,507.41
		**Low**	8,298.20	12,953.80
**Other HIC**	**0.03**	**High**	4,987.46	7,881.96
		**Low**	3,304.06	5,203.35
	**0.05**	**High**	4,667.06	6,541.63
		**Low**	3,091.80	4,316.87
	**0.07**	**High**	4,383.11	5,531.79
		**Low**	2,903.69	3,649.14
**MIC**	**0.03**	**High**	2,604.31	3,801.51
		**Low**	1,683.73	2,457.74
	**0.05**	**High**	2,417.15	3,045.89
		**Low**	1,562.73	1,969.22
	**0.07**	**High**	2,251.97	2,477.06
		**Low**	1,455.94	1,601.46
**GAVI**	**0.03**	**High**	983.84	786.55
		**Low**	550.95	440.47
	**0.05**	**High**	919.39	581.95
		**Low**	514.86	325.89
	**0.07**	**High**	862.34	434.61
		**Low**	482.91	243.38
**Indonesia and India**	**0.03**	**High**	1,807.90	1,258.95
		**Low**	1,136.10	791.14
	**0.05**	**High**	1,669.09	906.87
		**Low**	1,048.87	569.89
	**0.07**	**High**	1,545.54	658.69
		**Low**	971.23	413.93
**China**	**0.03**	**High**	4,342.43	2,938.32
		**Low**	2,884.17	1,951.58
	**0.05**	**High**	3,902.78	2,048.96
		**Low**	2,592.16	1,360.88
	**0.07**	**High**	3,517.86	1,440.22
		**Low**	2,336.50	956.57
**TOTAL**	**0.03**	**High**	28,936.17	44,252.10
		**Low**	19,001.38	29,163.92
	**0.05**	**High**	26,872.81	36,135.28
		**Low**	17,646.20	23,823.28
	**0.07**	**High**	25,049.13	30,049.77
		**Low**	16,448.47	19,818.28

*Source*: Own calculations based on S9 Table in S1 File.

### 3.2 Fixed costs

We next derive the PDV of fixed costs by applying Eq No. 3. Table 5 lists the estimated annual manufacturing fixed costs. These are based on the labor compensation for the required number of directors, managers and supervisors for the current factory in Durham, North Carolina, as presented in Clendinen *et al*. [12] and on the “factory and administrative overhead”, corresponding to 45% of the total cost for personnel and material (Section 2.3).

**Table 5:** Annual manufacturing fixed costs, in million 2018 US$.

	Low estimate	High estimate
Fixed costs for directors, managers, supervisors	1.000	1.436
Factory and administrative overhead	7.740	11.963
**Total fixed costs per factory per year (2007–2021)**	**8.740**	**13.399**
**Total fixed costs in 3 factories per year (2022–2028)**	**26.221**	**40.196**

*Source*: Own calculations displayed in S10a and S10b Table in S1 File.

Recently, Merck announced an expansion of the North Carolina facility and build a new one, the two facilities are expected to be fully operational from 2022 [81]. Since the additional factories will also be based in the U.S., we assume that the costs are the same in these additional factories. Thus, current fixed manufacturing costs are multiplied by three to obtain an estimate for fixed manufacturing costs from 2022 onwards.

Based on DTCA spending data for the Gardasil vaccines in the U.S. (90% of global DTCA spending), the assumptions on marketing spending on health providers, and the annual fixed manufacturing costs in Tables 5 and 6 displays the PDV of the stream of fixed costs and its split for the periods 2020–2028 and 2007–2028. The PDV of DTCA spending in the U.S. from 2007–2019 is in the range of US$ 582–659 million (in 2018 US$), depending on the interest rate.

**Table 6:** PDV of the stream of fixed manufacturing and marketing costs from 2020–2028 and 2007–2028, in million 2018 US$, applying Eq No. 3.

Interest rate (*r*)	Estimate	PDV fixed manufact. costs, 2020–28	PDV marketing costs, 2020–28	Total 2020–2028	PDV fixed manufact. costs, 2007–28	PDV of marketing costs, 2007–28	Total 2007–2028
**0.03**	**High**	269.548	123.240	**392.788**	330.320	2,118.761	**2,449.082**
	**Low**	175.833	74.032	**249.865**	215.476	1,272.781	**1,488.257**
**0.05**	**High**	247.675	114.688	**362.363**	263.503	1,970.337	**2,233.840**
	**Low**	161.565	68.895	**230.460**	171.890	1,183.620	**1,355.509**
**0.07**	**High**	228.379	107.129	**335.508**	214.590	1,844.744	**2,059.335**
	**Low**	148.977	64.354	**213.332**	139.983	1,108.174	**1,248.156**

*Source*: Own calculations based on Table 5 and S11 Table in S1 File.

Assuming that global marketing spending on health providers is 50% and 150% of global DTCA spending for the “low” and “high” estimate, respectively, implies that the PDV of the stream of global marketing costs from the perspective of 2007 is in the range of US$ 1.11–2.12 billion (in 2018 US$). It exceeds the PDV of manufacturing fixed costs for the period 2007–2028 considerably (US$ 140–330 million) and is at least as high as R&D costs, as will become apparent in Section 3.4. We estimate that the PDV of future manufacturing costs from the perspective of 2020 amounts to US$ 149–270 million. It exceeds the PDV of the stream of marketing costs in the period 2020–2028 (based on the annual average in the period 2017–2019), reflecting that marketing spending has sharply declined some years after market introduction. The PDV of total future fixed costs from the perspective of 2020 is in the range of US$ 213–393 million. For the entire patent length, the PDV of the total fixed cost stream from the perspective of 2007 amounts to US$ 1.25–2.45 billion.

### 3.3 Estimated global value of Gardasil patents and the patent buyout price

Using Eq No. 4, Table 7 shows the patent value from 2007–2028 and for the remaining patent length, 2020–2028, based on the information the PDV of the stream of operating profits and fixed costs, respectively. The PDV of the future profit stream in Table 7 is somewhat lower than shown in Table 4 as we left out the PDV of the operating profit stream from GAVI supported countries. The reason is that these countries are not obliged to provide patent protection on vaccines until 2033 [62] such that a patent buyout is not beneficial. We include those profits to derive the total patent value for 2007–2028. We estimate the global patent buyout price in 2020 when assuming patent expiry in 2028 in the range of US$ 15.6–27.7 billion (in 2018 US$), whereas the total patent value from the perspective of 2007 amounts to US$ 17.8–42.8 billion (in 2018 US$).

**Table 7:** Estimated global patent buyout price in 2020 and total patent value of Gardasil (2007–2028) in million 2018 US$, applying Eq No. 4.

Interest rate (*r*)	Patent value estimate*	PDV of operating profits, 2020–28**	PDV of fixed costs, 2020–28	Global patent buyout Price	PDV of operating profits, 2007–28	PDV of fixed costs, 2007–28	Total patent value
**0.03**	**High**	27,952.33	249.87	**27,702.46**	44,252.10	1’488.26	**42,763.84**
	**Low**	18,450.43	392.79	**18,057.64**	29,163.92	2’449.08	**26,714.84**
**0.05**	**High**	25,953.42	230.46	**25,722.96**	36,135.28	1’355.51	**34,779.77**
	**Low**	17,131.34	362.36	**16,768.97**	23,823.28	2’233.84	**21,589.44**
**0.07**	**High**	24,186.80	213.33	**23,973.46**	30,049.77	1’248.16	**28,801.62**
	**Low**	15,965.56	335.51	**15,630.06**	19,818.28	2’059.33	**17,758.94**

* For the high patent value estimate, we take the high estimate for the PDV of operating profits and deduct the low estimate of the PDV of fixed costs. Vice versa for the low patent value estimate.

** We did not include operating profits from GAVI supported countries.

*Source*: Own calculations based on S9 Table in S1 File and Table 6.

### 3.4 R&D cost estimates for Gardasil and its relation to the patent value

We next relate the total value of the Gardasil patents in Table 7 to its R&D costs and discuss the result in view of the observed POS for finding new vaccines in the literature.

In phase I of clinical trials the vaccine is tested in a small number of healthy individual to identify the best route to administer the vaccine, frequency and dose escalation, the maximum tolerated dose (MTD), and side effects [82]. The main aim of phase II is to demonstrate the efficacy and immunogenicity of the vaccine candidate in a larger group [82]. In phase III, the safety, immunogenicity, and efficacy of the final dosage of the vaccine is tested in thousands of subjects and is tested against a placebo and/or another vaccine in the market [59].

Light *et al*. [15] estimated that the cost per subject (set-up, recruitment, administration and support) is between US$ 100–400 in phase I, US$ 300–400 in phase II, and US$ 2000–3000 in phase III (in 2008 US$). Moreover, according to Sertkaya *et al*. [21], the site costs are US$ 682,284 for phase I, US$ 3,791,310 for phase II, and US$ 5,647,045 for phase III. The study costs for phase I is US$ 2,058,396 US$ 6,273,284 for phase II, and US$ 9,063,763 for phase III (average costs from 2004–2012 in current US$). We use these numbers jointly with the information of clinical trials (that includes subject numbers) to come up with R&D costs, separated by phases and type of cost.

As shown in Table 8, for Gardasil-4, we arrive at R&D costs in the range of US$ 539.80–594.41 million and for Gardasil-9 in the range of US$ 419.18–458.24 million.

**Table 8:** Estimated R&D costs on subjects, site and study of Gardasil-4 and Gardasil-9 (in 2018 US$).

		Estimate (in 2018 US$)			
		Gardasil-4		Gardasil-9	
		Low	High	Low	High
**Phase-I**	Total spent on subjects	94,070	376,279	18,372	73,488
	Total spent on sites	3,173,414	3,173,414	793,353	793,353
	Total spent of study costs	9,573,935	9,573,935	2,393,484	2,393,484
	Total spent on phase I clinical trials	12,841,419	13,123,628	3,202,637	3,250,037
**Phase-II**	Total spent on subjects	1,684,186	2,245,581	1,068,488	1,424,651
	Total spent on sites	22,042,500	22,042,500	17,634,000	17,634,000
	Total spent of study costs	36,472,581	36,472,581	29,178,065	29,178,065
	Total spent on phase II clinical trials	60,199,267	60,760,663	47,880,553	48,236,716
**Phase-III**	Total spent on subjects	107,539,535	161,309,302	77,297,674	115,946,512
	Total spent on sites	137,892,959	137,892,959	111,627,634	111,627,634
	Total spent of study costs	221,324,445	221,324,445	179,167,408	179,167,408
	Total spent on phase III clinical trials	466,756,940	520,526,707	368,092,716	406,741,553
	**Total spent on clinical trials**	539,797,626	594,410,998	419,178,479	458,238,595

*Source*: Own calculations based on S12 and S13 Tables in S1 File jointly with S14a Table in S1 File for Gardasil-4 and S14b Table in S1 File for Gardasil-9.

Table 9 presents total R&D costs by adding up the documented outlays for Gardasil-4 and Gardasil-9 and R&D capital costs. Capital costs are assumed to be 10% and 15% of the low estimate and high estimate of the R&D costs presented in Table 8, respectively. Doing so, we estimate total R&D costs of both vaccines between US$ 1,054.9–1,210.5 million.

**Table 9:** Total R&D cost derivation (in 2018 US$).

	Low estimate	High estimate
Gardasil-4 (subjects, site, study)	539,797,626	594,410,998
Gardasil-9 (subjects, site, study)	419,178,479	458,238,595
**Subtotal (subjects, site, study)**	**958,976,105**	**1,052,649,593**
Capital costs*	95,897,610	157,897,439
**Total Gardasil R&D costs**	**1,054,873,715**	**1,210,547,032**

* 10% of Subtotal for the low estimate, 15% of the Subtotal for the high estimate.

*Source*: Own calculations based on Table 8.

We finally put our estimates for total R&D costs in relation to the estimates of the total value of the Gardasil patents. This provides us with a range for the innovation probability that is required to cover R&D costs in expected value of the patents, as defined by *μ*_0_ in Eq No. 5. The lower bound is found by dividing the low estimate for R&D costs from Table 9 (US$ 1,055 million) and the high estimate of the patent value (US$ 42,764 million) from Table 7, which gives us *μ*_0_ = 0.025. For the upper bound, dividing the high estimate for R&D costs (US$ 1,210 million) and the low estimate of the patent value (US$ 17,759 million) implies *μ*_0_ = 0.068.

What do we know about the POS for vaccines targeting infectious diseases to which we can compare our estimate for *μ*_0_? In a sample of more than 1,800 trials at phase I in the 2000s, Wong *et al*. [83] found a POS of about one third. In the previous literature, the lowest POS estimates from moving from phase I to phase II and from moving to phase II to phase III are 50% and 22%, respectively, whereas the highest POS estimates are 90% and 79%, respectively [84]. This suggests a cumulative POS in clinical trials above 10%. Notably, the low POS estimates sometimes reported in the literature include pre-clinical trials that are not applicable here [85]. In sum, the POS estimates for other vaccines are significantly higher than our estimated *μ*_0_, suggesting that the Gardasil patents generate extraordinarily high profits.

## 4. Concluding remarks

Patents are an effective strategy to prevent new entrants in the market. The high number of patent applications for HPV vaccines by Merck and GSK in emerging countries with a large pharmaceutical industry such as China, Brazil and India shows that it is a strategy commonly implemented [60]. Patent buyouts for pharmaceuticals are potentially welfare-enhancing by removing monopoly price distortions and increasing drug availability by stimulating generic entry. Maintaining innovation incentives requires that the patent holder is fully compensated for its foregone profits. In this paper, we have calculated this patent buyout price for Merck’s HPV vaccines as the PDV of the future profit stream until the patent expiry in 2028.

We estimated that the remaining patent buyout value for Gardasil supplied by Merck in 2020 as the PDV of the future stream of expected profits in the range of US$ 15.6–27.7 billion (in 2018 US$), depending on the assumed interest rate for discounting and the estimated ranges for the number of demanded doses, variable costs, and fixed costs. On the one hand, the estimated remaining private patent value may be viewed as an upper bound for the patent buyout price since the original manufacturer Merck would still be able to make profits after the patent buyout because of its brand name and its established production capacity. On the other hand, given the private information of the patent holder, we may want to propose a sufficiently high patent buyout price that is unlikely to fall short of the remaining patent value, to incentivize the patent holder to agree to the patent buyout. In sum, albeit calculating the net social benefit of the patent buyout is beyond the scope of this paper, the suggested range for the patent buyout price may be viewed as a benchmark for negotiating an agreement with the patent holder.

While the global patent buyout of Gardasil we suggest is incentive-compatible for the patent holder, it is a financially challenging proposal. A preferable albeit not necessarily successful strategy to reduce prices could be to reach a licensing agreement with Merck to allow generic manufacturing of Gardasil for LIC and MIC. The Medicines Patent Pool (MPP), a United Nations-backed global health organization, negotiates with pharmaceutical companies and other global health stakeholders (i.e. governments and NGOs) licensing to allow generic companies to manufacture low cost medicines for LIC and MIC [86]. This patent pooling model has been successfully implemented for medicines against tuberculosis, HIV and hepatitis: 13 HIV antiretrovirals, three Hepatitis C direct-acting antivirals and one tuberculosis treatment have been licensed through MPP [86]. Merck licensed Raltegravir through MPP, a pediatric HIV antiretroviral medicine, for generic production for LIC and MIC [87]. However, this licensing agreement does not cover technology and data owned by Merck and in the case of Gardasil such an agreement has not been reached yet. The main obstacle to the MPP solution could be the concern of patent holders that entry of generic manufacturers may affect the market in HIC by parallel imports or a public debate on prices charged by the original manufacturer.

In order to assess the price setting power of Merck in providing its HPV vaccines, we have also estimated both the R&D costs and the global patent value, which is the PDV of the profit stream for the period 2007–2028. The ratio of these figures is the POS needed to break even in expected value. Using information on clinical trials, we estimated the R&D costs for the Gardasil innovation to be around US$ 1.05–1.21 billion, while the global patent value amounts to US$ 17.8–42.8 billion. The implied R&D to patent value ratio of 2.5–6.8% is below the average POS in clinical trials for vaccines found in the literature. As a caveat and suggestion for future research, making the point that the current patent system generates excessive price setting power, i.e. is more generous than needed to elicit desirable R&D effort, would require estimations of the relationship between R&D cost and the patent value for many more pharmaceuticals. Availability of such information may alter price negotiations for prescription drugs in health systems and may ultimately call for adjustment of the patent system.

## Supporting information

S1 File (DOCX)
