# Supplementary material for: The patent buyout price for human papilloma virus (HPV) vaccine and the ratio of R&D costs to the patent value
Source: PLoS One. 2021 Jan 11;16(1):e0244722. doi: 10.1371/journal.pone.0244722 (PMC7799842; doi:10.1371/journal.pone.0244722)
Supplement: S1 File — (DOCX) [file pone.0244722.s001.docx]

**The Patent Buyout Price for Human Papilloma Virus (HPV) Vaccine and the Ratio of R&D Costs to the Patent Value**

Throughout the paper, we converted all monetary variable values to 2018 US$, according to Table S0.

**Table S0.** Consumer price index (CPI) with base year 2015 and price index (PI) with base year 2018.

| **Observation date** | **CPI** | **PI*** |
| --- | --- | --- |
| 2007 | 87.48 | 0.826 |
| 2008 | 90.84 | 0.857 |
| 2009 | 90.52 | 0.854 |
| 2010 | 92.00 | 0.868 |
| 2011 | 94.90 | 0.896 |
| 2012 | 96.87 | 0.914 |
| 2013 | 98.29 | 0.928 |
| 2014 | 99.88 | 0.942 |
| 2015 | 100.00 | 0.944 |
| 2016 | 101.26 | 0.956 |
| 2017 | 103.42 | 0.976 |
| 2018 | 105.94 | 1.00 |

* PI is given by CPI (base year 2015) in a given year divided by CPI in 2018.

*Source:* Federal Reserve Bank [69] and own calculations.

To derive operating profits using equation No. 1, we need prices, number of sold units, and variable costs.

**Table S1.** Prices of Gardasil-4 and Gardasil-9 outside the U.S. in year 2016.*

|  | **2016, in current US$** | | **In 2018 US$**** | |
| --- | --- | --- | --- | --- |
|  | **Gardasil-4** | **Gardasil-9** | **Gardasil-4** | **Gardasil-9** |
| **HIC (excl. the U.S.)** | 44.12 | 91.00 | 45.96 | 94.79 |
| **MIC** | 19.70 | n.a. | 20.52 | n.a. |
| **GAVI** | 4.50 | n.a. | 4.69 | n.a. |
| **India** | 6.90 | n.a. | 7.19 | n.a. |
| **Indonesia** | 14.76 | n.a. | 15.38 | n.a. |
| **China** | 120.00 | 153.00 | 125.00 | 159.38 |

* Median prices for middle-income countries (MIC) and high-income countries (HIC). ** Deflated prices using the price index for 2016, PI=0.956 (Table S0). n.a.: Not applicable because Gardasil-9 has not been sold in these regions.

*Sources:* HIC, MIC (includes purchases mediated by PAHO), GAVI [9]; India [75]; Indonesia [76]; China [73,74] and own calculations.

**Table S2.** Gardasil prices* per dose in the U.S. from 2007-2018.

| **Year** | **CDC (in current US$)**** | **Private Sector (in current US$)** | **Average price (in current US$)***** | **Estimated Price (in 2018 US$)****** |
| --- | --- | --- | --- | --- |
| **2007** | 96.75 | 120.50 | 108.63 | 131.55 |
| **2008** | 100.59 | 125.29 | 112.94 | 131.72 |
| **2009** | 105.58 | 130.27 | 117.93 | 138.03 |
| **2010** | 108.72 | 130.27 | 119.50 | 137.61 |
| **2011** | 95.75 | 130.27 | 113.01 | 126.16 |
| **2012** | 98.60 | 135.45 | 117.03 | 127.99 |
| **2013** | 107.16 | 135.45 | 121.30 | 130.76 |
| **2014** | 121.03 | 141.38 | 131.21 | 139.17 |
| **2015** | 121.03 | 160.17 | 140.60 | 148.96 |
| **2016** | 119.04 | 193.63 | 156.34 | 163.57 |
| **2017** | 116.22 | 204.87 | 160.55 | 164.47 |
| **2018** | 144.18 | 217.11 | 180.64 | 180.64 |

* Prices for Gardasil-4 in 2007-2015 and for Gardasil-9 for 2016-2018. **CDC refers to prices for sales mediated by Centers for Disease Control (CDC) funded programs. *** Simple average of numbers in columns 2 and 3. **** Deflated by the PI as given in Table S0.

*Source:* CDC [40] and own calculations.

GAVI [19] gives estimates on the number of units for 2010-2017 and predictions for 2018-2027 for HIC as a whole and for other regions. The number of units is thus not readily available as breakdown of HIC numbers into those sold in the U.S. and those sold in other HIC. We also do not have estimates for the period 2007-2009. We can derive, however, the market share for the period 2015-2017 in the U.S. and know that in period 2007-2009 Gardasil has only been sold in HIC. Using revenue information in Table 1 of the main text, the missing data to generate the middle estimates for 2007-2017 in Table 2 (“Number of Gardasil doses sold in different countries and country groups…”) is derived accordingly in Tables S3a-S3c.

**Table S3a.** U.S. market share, 2015-2017.

| **Year** | **U.S. revenue (in million US$)** | **Total revenue (in million US$)** | **U.S. revenue share (in %)** |
| --- | --- | --- | --- |
| **2015** | 1,520 | 1,908 | 79.66 |
| **2016** | 1,780 | 2,173 | 81.91 |
| **2017** | 1,565 | 2,308 | 67.81 |
| **Average U.S revenue share 2015-2017 (in %)** | | | 76.46 |

*Source:* Merck [77].

**Table S3b.** Total Gardasil sales revenues, its estimated composition (U.S. vs. non-U.S.) and estimated number of doses sold in the U.S., 2007-2017.

|  | **Revenue (in million US$)** | | |  | |
| --- | --- | --- | --- | --- | --- |
| **Year** | **U.S.*** | **Non-U.S.*** | **Total** | **Nominal U.S. price/dose**** | **Number of doses sold in the U.S. (in millions)** |
| **2007** | 1,132.07 | 348.53 | 1,480.60 | 108.63 | 10.42 |
| **2008** | 1,072.58 | 330.22 | 1,402.80 | 112.94 | 9.50 |
| **2009** | 855.13 | 263.27 | 1,118.40 | 117.93 | 7.25 |
| **2010** | 755.42 | 232.58 | 988 | 119.5 | 6.32 |
| **2011** | 924.40 | 284.60 | 1,209 | 113.01 | 8.18 |
| **2012** | 1,247.06 | 383.94 | 1,631 | 117.03 | 10.66 |
| **2013** | 1,399.98 | 431.02 | 1,831 | 121.30 | 11.54 |
| **2014** | 1,328.87 | 409.13 | 1,738 | 131.21 | 10.13 |
| **2015** | 1,520 | 388 | 1,908 | 140.60 | 10.81 |
| **2016** | 1,780 | 393 | 2,173 | 156.34 | 11.39 |
| **2017** | 1,565 | 743 | 2,308 | 160.55 | 9.75 |

* For 2007-2014 we assumed the U.S. (non-U.S.) market share corresponds to 76.46% (23.54%) of the global Gardasil revenue market, as calculated in Table S3a, using total revenues from Table 1 (main text). ** The column restates the average median price in the U.S. from Table S2, assuming that 50% of sales are mediated by CDC funded programs.

*Sources:* GAVI [19] and own calculations.

**Table S3c.** Nominal prices and number of doses in HIC excluding the U.S., 2007-2009.

| **Year** | **Revenue**  **(in million US$)*** | **Nominal price/dose in other HIC (in US$)**** | **Number of doses sold in other HIC (in millions)***** |
| --- | --- | --- | --- |
| **2007** | 348.53 | 37.95 | 9.18 |
| **2008** | 330.22 | 39.41 | 8.38 |
| **2009** | 263.27 | 39.27 | 6.70 |

* Revenue information for non-U.S. countries from Table S3b (these are HIC in 2007-2009). ** Prices from Table S1 deflated by PI from Table S0. *** Revenue in column 2 divided by price in column 3.

For the forecasts (2018-2027) in Table 2 of the main text we employ the average U.S. market share in period 2010-2017 as derived in Table S4 for the breakdown of the number of units sold in the U.S. vs. other HIC.

**Table S4.** U.S. share of total number of doses sold in HIC, 2010-2017.

| **Year** | **Number of doses sold in all HIC (in millions)** | **Number of doses sold in U.S. (in millions)*** | **U.S. sales share (in %)** |
| --- | --- | --- | --- |
| **2010** | 15.66 | 6.32 | 40.4 |
| **2011** | 20.88 | 8.18 | 39.2 |
| **2012** | 18.27 | 10.66 | 58.3 |
| **2013** | 17.4 | 11.54 | 66.3 |
| **2014** | 15.23 | 10.13 | 66.5 |
| **2015** | 14.79 | 10.81 | 73.1 |
| **2016** | 15.66 | 11.39 | 72.7 |
| **2017** | 15.66 | 9.75 | 62.2 |
| **Average U.S. sales share 2010-2017 (in %)** | | | **59.8** |

* Calculated in Table S3b.

*Source:* GAVI [19] and own calculations.

Table S5a and Table S5b summarize the derived middle estimates of the number of doses for the periods 2007-2017 and 2018-2027 (forecasts), respectively: they also display the “high” estimates (adding 20% to the middle estimate) and the “low” estimates (subtracting 20% from the middle estimate) that account for uncertainty in the estimates and predictions. The “high” and “low” estimates by region enter the calculations of operating profits (Table S9). From those, we can derive the PDV of operating profits for the two scenarios as given in Table 4 of the main text by applying equation No. 2.

**Table S5a.** Number of Gardasil doses in different regions in millions, 2007-2017, different scenarios.

| **Year** | **Estimate** | **Number of doses (in millions)** | | | | | | | |
| --- | --- | --- | --- | --- | --- | --- | --- | --- | --- |
|  |  | **HIC total** | **U.S.*** | **Other HIC**** | **MIC** | **GAVI** | **India/**  **Indonesia** | **China** | **Total** |
| **2007** | Low (-20%) | 15.68 | 8.34 | 7.34 | 0 | 0 | 0 | 0 | 15.68 |
|  | Middle | 19.60 | 10.42 | 9.18 | 0 | 0 | 0 | 0 | 19.60 |
|  | High (+20%) | 23.52 | 12.50 | 11.02 | 0 | 0 | 0 | 0 | 23.52 |
| **2008** | Low (-20%) | 14.30 | 7.60 | 6.70 | 0 | 0 | 0 | 0 | 14.30 |
|  | Middle | 17.88 | 9.50 | 8.38 | 0 | 0 | 0 | 0 | 17.88 |
|  | High (+20%) | 21.46 | 11.40 | 10.06 | 0 | 0 | 0 | 0 | 21.46 |
| **2009** | Low (-20%) | 11.16 | 5.80 | 5.36 | 0 | 0 | 0 | 0 | 11.16 |
|  | Middle | 13.95 | 7.25 | 6.70 | 0 | 0 | 0 | 0 | 13.95 |
|  | High (+20%) | 16.74 | 8.70 | 8.04 | 0 | 0 | 0 | 0 | 16.74 |
| **2010** | Low (-20%) | 12.53 | 5.06 | 7.47 | 4.87 | 0 | 0 | 0 | 17.40 |
|  | Middle | 15.66 | 6.32 | 9.34 | 6.09 | 0 | 0 | 0 | 21.75 |
|  | High (+20%) | 18.79 | 7.59 | 11.21 | 7.31 | 0 | 0 | 0 | 26.10 |
| **2011** | Low (-20%) | 16.70 | 6.54 | 10.16 | 9.05 | 0 | 0 | 0 | 25.75 |
|  | Middle | 20.88 | 8.18 | 12.70 | 11.31 | 0 | 0 | 0 | 32.19 |
|  | High (+20%) | 25.06 | 9.82 | 15.24 | 13.57 | 0 | 0 | 0 | 38.63 |
| **2012** | Low (-20%) | 14.62 | 8.53 | 6.09 | 11.14 | 0 | 0 | 0 | 25.75 |
|  | Middle | 18.27 | 10.66 | 7.61 | 13.92 | 0 | 0 | 0 | 32.19 |
|  | High (+20%) | 21.92 | 12.79 | 9.14 | 16.70 | 0 | 0 | 0 | 38.63 |
| **2013** | Low (-20%) | 13.92 | 9.23 | 4.69 | 11.14 | 0.70 | 0 | 0 | 25.75 |
|  | Middle | 17.40 | 11.54 | 5.86 | 13.92 | 0.87 | 0 | 0 | 32.19 |
|  | High (+20%) | 20.88 | 13.85 | 7.03 | 16.70 | 1.04 | 0 | 0 | 38.63 |
| **2014** | Low (-20%) | 12.18 | 8.10 | 4.08 | 9.05 | 0.70 | 0 | 0 | 21.92 |
|  | Middle | 15.23 | 10.13 | 5.10 | 11.31 | 0.87 | 0 | 0 | 27.41 |
|  | High (+20%) | 18.27 | 12.15 | 6.12 | 13.57 | 1.04 | 0 | 0 | 32.89 |
| **2015** | Low (-20%) | 11.83 | 8.65 | 3.18 | 7.66 | 1.39 | 0 | 0 | 20.88 |
|  | Middle | 14.79 | 10.81 | 3.98 | 9.57 | 1.74 | 0 | 0 | 26.10 |
|  | High (+20%) | 17.75 | 12.97 | 4.78 | 11.48 | 2.09 | 0 | 0 | 31.32 |
| **2016** | Low (-20%) | 12.53 | 9.11 | 3.42 | 7.66 | 2.09 | 0.70 | 0 | 22.97 |
|  | Middle | 15.66 | 11.39 | 4.27 | 9.57 | 2.61 | 0.87 | 0 | 28.71 |
|  | High (+20%) | 18.79 | 13.66 | 5.13 | 11.48 | 3.13 | 1.04 | 0 | 34.45 |
| **2017** | Low (-20%) | 12.53 | 7.80 | 4.73 | 7.66 | 1.39 | 0.70 | 0 | 22.27 |
|  | Middle | 15.66 | 9.75 | 5.91 | 9.57 | 1.74 | 0.87 | 0 | 27.84 |
|  | High (+20%) | 18.79 | 11.70 | 7.09 | 11.48 | 2.09 | 1.04 | 0 | 33.41 |
| **Total** | **Low (-20%)** | 106.84 | **63.02** | **43.82** | **68.21** | **6.26** | **1.39** | **0** | **182.70** |
|  | **Middle** | 133.55 | **78.77** | **54.77** | **85.26** | **7.83** | **1.74** | **0** | **228.38** |
|  | **High (+20%)** | 160.25 | **94.53** | **65.73** | **102.31** | **9.40** | **2.09** | **0** | **274.05** |

* For the U.S. middle estimates, see Table S3b. ** Implied by subtracting U.S. numbers from HIC total.

**Table S5b.** Number of Gardasil doses in different regions in millions, 2018-2027, different scenarios.

| **Year** | **Estimate** | **Number of doses (in millions)** | | | | | | | |
| --- | --- | --- | --- | --- | --- | --- | --- | --- | --- |
|  |  | **HIC total** | **U.S.*** | **Other HIC**** | **MIC** | **GAVI** | **India/**  **Indonesia** | **China** | **Total** |
| **2018** | Low (-20%) | 11.83 | 7.08 | 4.76 | 8.35 | 4.18 | 0.70 | 0 | 25.06 |
|  | Middle | 14.79 | 8.84 | 5.95 | 10.44 | 5.22 | 0.87 | 0 | 31.32 |
|  | High (+20%) | 17.75 | 10.61 | 7.13 | 12.53 | 6.26 | 1.04 | 0 | 37.58 |
| **2019** | Low (-20%) | 12.18 | 7.28 | 4.90 | 9.74 | 18.79 | 0.70 | 0 | 41.41 |
|  | Middle | 15.23 | 9.10 | 6.12 | 12.18 | 23.49 | 0.87 | 0 | 51.77 |
|  | High (+20%) | 18.27 | 10.93 | 7.34 | 14.62 | 28.19 | 1.04 | 0 | 62.12 |
| **2020** | Low (-20%) | 13.22 | 7.91 | 5.32 | 10.44 | 27.84 | 0.70 | 0 | 52.20 |
|  | Middle | 16.53 | 9.88 | 6.65 | 13.05 | 34.80 | 0.87 | 0 | 65.25 |
|  | High (+20%) | 19.84 | 11.86 | 7.97 | 15.66 | 41.76 | 1.04 | 0 | 78.30 |
| **2021** | Low (-20%) | 12.53 | 7.49 | 5.04 | 10.44 | 21.58 | 11.14 | 0 | 55.68 |
|  | Middle | 15.66 | 9.36 | 6.30 | 13.05 | 26.97 | 13.92 | 0 | 69.60 |
|  | High (+20%) | 18.79 | 11.24 | 7.55 | 15.66 | 32.36 | 16.70 | 0 | 83.52 |
| **2022** | Low (-20%) | 11.83 | 7.08 | 4.76 | 10.44 | 22.97 | 20.18 | 0.70 | 66.12 |
|  | Middle | 14.79 | 8.84 | 5.95 | 13.05 | 28.71 | 25.23 | 0.87 | 82.65 |
|  | High (+20%) | 17.75 | 10.61 | 7.13 | 15.66 | 34.45 | 30.28 | 1.04 | 99.18 |
| **2023** | Low (-20%) | 11.14 | 6.66 | 4.48 | 10.44 | 25.06 | 18.79 | 2.09 | 67.51 |
|  | Middle | 13.92 | 8.32 | 5.60 | 13.05 | 31.32 | 23.49 | 2.61 | 84.39 |
|  | High (+20%) | 16.70 | 9.99 | 6.72 | 15.66 | 37.58 | 28.19 | 3.13 | 101.27 |
| **2024** | Low (-20%) | 10.44 | 6.24 | 4.20 | 11.14 | 17.40 | 19.49 | 2.78 | 61.25 |
|  | Middle | 13.05 | 7.80 | 5.25 | 13.92 | 21.75 | 24.36 | 3.48 | 76.56 |
|  | High (+20%) | 15.66 | 9.36 | 6.30 | 16.70 | 26.10 | 29.23 | 4.18 | 91.87 |
| **2025** | Low (-20%) | 9.74 | 5.83 | 3.92 | 11.83 | 22.27 | 17.40 | 2.78 | 64.03 |
|  | Middle | 12.18 | 7.28 | 4.90 | 14.79 | 27.84 | 21.75 | 3.48 | 80.04 |
|  | High (+20%) | 14.62 | 8.74 | 5.88 | 17.75 | 33.41 | 26.10 | 4.18 | 96.05 |
| **2026** | Low (-20%) | 9.74 | 5.83 | 3.92 | 11.83 | 20.88 | 16.01 | 3.48 | 61.94 |
|  | Middle | 12.18 | 7.28 | 4.90 | 14.79 | 26.10 | 20.01 | 4.35 | 77.43 |
|  | High (+20%) | 14.62 | 8.74 | 5.88 | 17.75 | 31.32 | 24.01 | 5.22 | 92.92 |
| **2027** | Low (-20%) | 9.74 | 5.83 | 3.92 | 11.83 | 20.18 | 14.62 | 4.87 | 61.25 |
|  | Middle | 12.18 | 7.28 | 4.90 | 14.79 | 25.23 | 18.27 | 6.09 | 76.56 |
|  | High (+20%) | 14.62 | 8.74 | 5.88 | 17.75 | 30.28 | 21.92 | 7.31 | 91.87 |
| **Total** | **Low (-20%)** | 112.40 | **67.22** | **45.19** | **106.49** | **201.14** | **119.71** | **16.70** | **556.45** |
|  | **Middle** | 140.51 | **84.02** | **56.48** | **133.11** | **251.43** | **149.64** | **20.88** | **695.57** |
|  | **High (+20%)** | 168.61 | **100.83** | **67.78** | **159.73** | **301.72** | **179.57** | **25.06** | **834.68** |

* Assuming for the middle estimates that number doses sold in the U.S. corresponds to 59.8% of total number of doses sold in HIC markets (see Table S4). ** Implied by subtracting U.S. numbers from HIC total.

*Source:* GAVI [19] and own calculations.

We next come to variable costs that are composed of capital costs, costs for materials, costs for filling and packaging, and labor costs. Based on variable costs, the price information presented in Tables S1 and S2, and the number of units in Tables S5a and S5b, we can apply equation No. 1 to compute operating profits, and accordingly equation No. 2 to compute the PDV of the stream of operating profits.

We first restate in Table S6a the calculation of Clendinen *et al.* [12] who annualize capital costs by assuming a 5% real (no inflation) discount rate and a useful life of 10 years for equipment and 25 years for building. The range of costs reflects uncertainty in estimates. In Table S6b we obtain variable capital costs by dividing the total annualized capital costs by the average number of doses per year produced in the period 2010-2017, using the middle estimates in Table 5a.

**Table S6a.** Total investment costs and annualized capital costs for Gardasil-4, in million 2014 US$.

|  | **Low Estimate** | **High Estimate** |
| --- | --- | --- |
| **Investment** | | |
| Building cost | 100 | 166.7 |
| Equipment cost | 50 | 83.3 |
| **Total investment in building and equipment** | 150 | 250 |
| **Cost of capital** | | |
| Annualized capital cost - Building | 6.8 | 11.3 |
| Annualized capital cost - Equipment | 6.2 | 10.3 |
| **Total annualized capital cost** | 13 | 21.6 |

*Source*: Clendinen *et al.* [12].

**Table S6b.** Estimated variable capital costs (per year) for manufacturing one million doses of Gardasil-4**.**

|  | **Low estimate** | **High estimate** |
| --- | --- | --- |
| Variable building cost (annualized capital cost/28.55 million)* | 238,204.71 | 395,840.18 |
| Variable equipment cost (annualized capital cost/28.55 million)* | 217,186.64 | 360,810.07 |
| **Total variable capital cost for one million doses (in 2014 US$)** | 455,391.35 | 756,650.25 |
| **Total variable capital cost for one million doses (in 2018 US$)**** | 428,067.87 | 711,251.24 |

* To obtain variable costs per million doses, variable capital costs from Table S6a are divided by the average number of doses sold per year between 2010-2017 (i.e. 228.38/8=28.55 million, according to middle estimates in Table S5a). ** Numbers in row 3 deflated by price index for 2014, PI=0.942 (Table S0).

*Source:* Own calculations.

Costs for materials are derived in Table S7, whereas Table S8 presents the estimates of variable labor costs. Table 3 of the main text shows total variable costs based on Tables S6b-S8.

**Table S7.** Estimated cost of materials for producing one million doses of Gardasil-4.

| **Materials (units)** | **Units per package sold by life science companies** | **Price per package (2013 US$)** | **Number of units needed per million doses** | **Price for one million doses (2013 US$)*** |
| --- | --- | --- | --- | --- |
| Yeast Media (kg) | 0.02 | 100 | 7.11 | 35,532.47 |
| Yeast Extract (kg) | 1.00 | 300 | 88.83 | 26,649.35 |
| Soy Protein (kg) | 25.00 | 2,600 | 44.42 | 4,619.22 |
| Magnesium Chloride (kg) | 5.00 | 150 | 0.78 | 23.38 |
| Thimerosal (g) | 500.00 | 2,000 | 18.17 | 72.67 |
| Glucose (kg) | 2.50 | 250 | 88.83 | 8,883.12 |
| Sodium Hydroxide (kg) | 5.00 | 200 | 4.04 | 161.45 |
| Galactose (kg) | 5.00 | 700 | 177.66 | 24,872.73 |
| PS-­‐80 (kg) | 25.00 | 360 | 10.09 | 145.37 |
| Sodium Chloride (kg) | 50.00 | 550 | 646.04 | 7,106.49 |
| DTT (g) | 100.00 | 1,000 | 38.96 | 389.61 |
| Benzonase (ku) | 25.00 | 180 | 299.81 | 2,158.60 |
| MOPS (kg) | 5.00 | 2,000 | 1.95 | 779.22 |
| Ammonium sulfate (kg) | 5.00 | 290 | 9.81 | 569.00 |
| Microfiltration filters | 1.00 | 10,000 | 0.10 | 974.03 |
| Hollow-­‐fiber membranes | 1.00 | 9,000 | 0.10 | 876.62 |
| PVDF—Millipore200 (3pk=3000L) | 1.00 | 850 | 0.10 | 82.79 |
| Poros 50HS beads 20x3ft (L) | 10.00 | 22,500 | 34.77 | 78,222.56 |
| Filling and packaging | | | | 310,000.00 |
| High Estimate – Total listed retail prices (in 2013 US$) | | | | **502,118.67** |
| **High Estimate – Total listed retail prices (in 2018 US$)**** | | | | **539,912.55** |
| **Low Estimate – Discounted at 40% (in 2018 US$)**** | | | | **323,947.53** |

* The price for one million doses in the last column 4 is obtained by multiplying the numbers in column 2 and 3 and dividing by the units per package in column 1 ** Deflated by price index for 2013, PI=0.928 (Table S0).

*Source*: Clendinen *et al.* [12] and own calculations.

According to Clendinen *et al.* [12], it takes 152 personnel across different functions (management, manufacturing, inspection and quality assurance) to manufacture two sets of batches or up to 30.8 million doses of Gardasil-4 in one year. Based on their estimates, we arrive at Table S8 for variable labor costs.

**Table S8.** Variable labor cost for producing one million doses of Gardasil-4.

| **Number of employees** | **Personnel** | **Salary costs per employee for one million doses (in 2014 US$)** | | **Variable labor costs (in 2014 US$) per million doses** | |
| --- | --- | --- | --- | --- | --- |
|  | **Type** | **Low** | **High** | **Low** | **High** |
| 60 | Manufacturing Operators | 1,623.38 | 2,272.73 | 97,402.60 | 136,363.64 |
| 47 | Quality Assurance and Quality Control Operators | 1,623.38 | 2,272.73 | 76,298.70 | 106,818.18 |
| 34 | Fill/Pack Staff | 1,623.38 | 2,272.73 | 55,194.81 | 77,272.73 |
| **Total (in 2014 US$)** | | | | 228,896.10 | 320,454.55 |
| **Total (in 2018 US$)*** | | | | 243,506.49 | 340,909.09 |

* Total variable costs deflated by the price index for 2014, PI=0.942 (Table S0).

*Source:* Clendinen *et al.* [12] and own calculations.

Table S9 uses prices, number of units, and variable costs to calculate operating profits.

**Table S9.** Detailed calculation for Table 4 of operating profits for different countries and country groups (a) 2007-2009, (b) 2010-2019 and (c) 2019-2028, applying equation No. 1.

1. Operating profits estimation, 2007-2009

|  |  |  | **2007** | **2008** | **2009** |
| --- | --- | --- | --- | --- | --- |
| **U.S.** | Price | | 131.55 | 131.72 | 138.03 |
|  | Variable costs | Low | 1.00 | 1.00 | 1.00 |
|  |  | High | 1.59 | 1.59 | 1.59 |
|  | # doses (in millions) | Low | 8.34 | 7.60 | 5.80 |
|  |  | High | 12.50 | 11.40 | 8.70 |
|  | Profit (in million 2018 US$) | Low | 1,083.37 | 989.00 | 791.33 |
|  |  | High | 1,632.43 | 1,490.23 | 1,192.13 |
| **Other HIC** | Price | | 45.96 | 45.96 | 45.96 |
|  | Variable costs | Low | 1.00 | 1.00 | 1.00 |
|  |  | High | 1.59 | 1.59 | 1.59 |
|  | # doses (in millions) | Low | 7.34 | 6.70 | 5.36 |
|  |  | High | 11.02 | 10.06 | 8.04 |
|  | Profit (in million 2018 US$) | Low | 325.84 | 297.45 | 237.81 |
|  |  | High | 495.26 | 452.10 | 361.47 |
| **Total profit (in million 2018 US$)** | | **Low** | **1,409.21** | **1,286.45** | **1,029.15** |
|  |  | **High** | **2,127.69** | **1,942.33** | **1,553.59** |

1. Operating profits estimation, 2010-2019

|  |  |  | **2010** | **2011** | **2012** | **2013** | **2014** | **2015** | **2016** | **2017** | **2018** | **2019** |
| --- | --- | --- | --- | --- | --- | --- | --- | --- | --- | --- | --- | --- |
| **U.S.** | Price | | 137.61 | 126.16 | 127.99 | 130.76 | 139.17 | 148.96 | 163.57 | 164.47 | 180.64 | 180.64 |
|  | Variable costs | Low | 1.00 | 1.00 | 1.00 | 1.00 | 1.00 | 1.00 | 1.00 | 1.00 | 1.00 | 1.00 |
|  |  | High | 1.59 | 1.59 | 1.59 | 1.59 | 1.59 | 1.59 | 1.59 | 1.59 | 1.59 | 1.59 |
|  | # doses (in millions) | Low | 5.06 | 6.54 | 8.53 | 9.23 | 8.10 | 8.65 | 9.11 | 7.80 | 7.08 | 7.28 |
|  |  | High | 7.59 | 9.82 | 12.79 | 13.85 | 12.15 | 12.97 | 13.66 | 11.70 | 10.61 | 10.93 |
|  | Profit (in million 2018 US$) | Low | 687.90 | 815.15 | 1,077.57 | 1,192.56 | 1,114.75 | 1,274.54 | 1,475.37 | 1,270.18 | 1,266.87 | 1,304.14 |
|  |  | High | 1,036.33 | 1,228.51 | 1,623.91 | 1,797.02 | 1,679.30 | 1,919.46 | 2,221.12 | 1,912.17 | 1,906.57 | 1,962.65 |
| **Other HIC** | Price | | 45.96 | 45.96 | 45.96 | 45.96 | 45.96 | 45.96 | 45.96 | 45.96 | 45.96 | 94.79 |
|  | Variable costs | Low | 1.00 | 1.00 | 1.00 | 1.00 | 1.00 | 1.00 | 1.00 | 1.00 | 1.00 | 1.00 |
|  |  | High | 1.59 | 1.59 | 1.59 | 1.59 | 1.59 | 1.59 | 1.59 | 1.59 | 1.59 | 1.59 |
|  | # doses (in millions) | Low | 7.47 | 10.16 | 6.09 | 4.69 | 4.08 | 3.18 | 3.42 | 4.73 | 4.76 | 4.90 |
|  |  | High | 11.21 | 15.24 | 9.14 | 7.03 | 6.12 | 4.78 | 5.13 | 7.09 | 7.13 | 7.34 |
|  | Profit (in million 2018 US$) | Low | 331.46 | 450.79 | 270.24 | 207.96 | 180.91 | 141.24 | 151.71 | 209.84 | 211.04 | 456.34 |
|  |  | High | 503.80 | 685.17 | 410.75 | 316.09 | 274.97 | 214.68 | 230.59 | 318.95 | 320.76 | 688.84 |
| **MIC** | Price | | 20.52 | 20.52 | 20.52 | 20.52 | 20.52 | 20.52 | 20.52 | 20.52 | 20.52 | 20.52 |
|  | Variable costs | Low | 1.00 | 1.00 | 1.00 | 1.00 | 1.00 | 1.00 | 1.00 | 1.00 | 1.00 | 1.00 |
|  |  | High | 1.59 | 1.59 | 1.59 | 1.59 | 1.59 | 1.59 | 1.59 | 1.59 | 1.59 | 1.59 |
|  | # doses (in millions) | Low | 4.87 | 9.05 | 11.14 | 11.14 | 9.05 | 7.66 | 7.66 | 7.66 | 8.35 | 9.74 |
|  |  | High | 7.31 | 13.57 | 16.70 | 16.70 | 13.57 | 11.48 | 11.48 | 11.48 | 12.53 | 14.62 |
|  | Profit (in million 2018 US$) | Low | 92.23 | 171.29 | 210.81 | 210.81 | 171.29 | 144.93 | 144.93 | 144.93 | 158.11 | 184.46 |
|  |  | High | 142.66 | 264.94 | 326.08 | 326.08 | 264.94 | 224.18 | 224.18 | 224.18 | 244.56 | 285.32 |
| **GAVI** | Price | |  |  |  | 4.69 | 4.69 | 4.69 | 4.69 | 4.69 | 4.69 | 4.69 |
|  | Variable costs | Low |  |  |  | 1.00 | 1.00 | 1.00 | 1.00 | 1.00 | 1.00 | 1.00 |
|  |  | High |  |  |  | 1.59 | 1.59 | 1.59 | 1.59 | 1.59 | 1.59 | 1.59 |
|  | # doses (in millions) | Low | 0.00 | 0.00 | 0.00 | 0.70 | 0.70 | 1.39 | 2.09 | 1.39 | 4.18 | 18.79 |
|  |  | High | 0.00 | 0.00 | 0.00 | 1.04 | 1.04 | 2.09 | 3.13 | 2.09 | 6.26 | 28.19 |
|  | Profit (in million 2018 US$) | Low | 0.00 | 0.00 | 0.00 | 2.16 | 2.16 | 4.31 | 6.47 | 4.31 | 12.94 | 58.21 |
|  |  | High | 0.00 | 0.00 | 0.00 | 3.85 | 3.85 | 7.70 | 11.55 | 7.70 | 23.10 | 103.94 |
| **Indonesia and India** | Price | |  |  |  |  |  |  | 11.28 | 11.28 | 11.28 | 11.28 |
|  | Variable costs | Low |  |  |  |  |  |  | 1.00 | 1.00 | 1.00 | 1.00 |
|  |  | High |  |  |  |  |  |  | 1.59 | 1.59 | 1.59 | 1.59 |
|  | # doses (in millions) | Low | 0.00 | 0.00 | 0.00 | 0.00 | 0.00 | 0.00 | 0.70 | 0.70 | 0.70 | 0.70 |
|  |  | High | 0.00 | 0.00 | 0.00 | 0.00 | 0.00 | 0.00 | 1.04 | 1.04 | 1.04 | 1.04 |
|  | Profit (in million 2018 US$) | Low | 0.00 | 0.00 | 0.00 | 0.00 | 0.00 | 0.00 | 6.75 | 6.75 | 6.75 | 6.75 |
|  |  | High | 0.00 | 0.00 | 0.00 | 0.00 | 0.00 | 0.00 | 10.73 | 10.73 | 10.73 | 10.73 |
| **Total profit (in million 2018 US$)** | | **Low** | **1,111.59** | **1,437.22** | **1,558.63** | **1,613.49** | **1,469.10** | **1,565.02** | **1,785.23** | **1,636.02** | **1,655.70** | **2,009.89** |
|  |  | **High** | **1,682.79** | **2,178.62** | **2,360.74** | **2,443.03** | **2,223.05** | **2,366.01** | **2,698.17** | **2,473.73** | **2,505.73** | **3,051.49** |

1. Operating profit estimation, 2020-2028

|  |  |  | **2020** | **2021** | **2022** | **2023** | **2024** | **2025** | **2026** | **2027/2028** |
| --- | --- | --- | --- | --- | --- | --- | --- | --- | --- | --- |
| **U.S.** | Price | | 180.64 | 180.64 | 180.64 | 180.64 | 180.64 | 180.64 | 180.64 | 180.64 |
|  | Variable costs | Low | 1.00 | 1.00 | 1.00 | 1.00 | 1.00 | 1.00 | 1.00 | 1.00 |
|  |  | High | 1.59 | 1.59 | 1.59 | 1.59 | 1.59 | 1.59 | 1.59 | 1.59 |
|  | # doses (in millions) | Low | 7.91 | 7.49 | 7.08 | 6.66 | 6.24 | 5.83 | 5.83 | 5.83 |
|  |  | High | 11.86 | 11.24 | 10.61 | 9.99 | 9.36 | 8.74 | 8.74 | 8.74 |
|  | Profit (in million 2018 US$) | Low | 1,415.92 | 1,341.40 | 1,266.87 | 1,192.35 | 1,117.83 | 1,043.31 | 1,043.31 | 1,043.31 |
|  |  | High | 2,130.88 | 2,018.73 | 1,906.57 | 1,794.42 | 1,682.27 | 1,570.12 | 1,570.12 | 1,570.12 |
| **Other HIC** | Price | | 94.79 | 94.79 | 94.79 | 94.79 | 94.79 | 94.79 | 94.79 | 94.79 |
|  | Variable costs | Low | 1.00 | 1.00 | 1.00 | 1.00 | 1.00 | 1.00 | 1.00 | 1.00 |
|  |  | High | 1.59 | 1.59 | 1.59 | 1.59 | 1.59 | 1.59 | 1.59 | 1.59 |
|  | # doses (in millions) | Low | 5.32 | 5.04 | 4.76 | 4.48 | 4.20 | 3.92 | 3.92 | 3.92 |
|  |  | High | 7.97 | 7.55 | 7.13 | 6.72 | 6.30 | 5.88 | 5.88 | 5.88 |
|  | Profit (in million 2018 US$) | Low | 495.46 | 469.38 | 443.30 | 417.23 | 391.15 | 365.07 | 365.07 | 365.07 |
|  |  | High | 747.89 | 708.53 | 669.16 | 629.80 | 590.44 | 551.08 | 551.08 | 551.08 |
| **MIC** | Price | | 20.52 | 20.52 | 20.52 | 20.52 | 20.52 | 20.52 | 20.52 | 20.52 |
|  | Variable costs | Low | 1.00 | 1.00 | 1.00 | 1.00 | 1.00 | 1.00 | 1.00 | 1.00 |
|  |  | High | 1.59 | 1.59 | 1.59 | 1.59 | 1.59 | 1.59 | 1.59 | 1.59 |
|  | # doses (in millions) | Low | 10.44 | 10.44 | 10.44 | 10.44 | 11.14 | 11.83 | 11.83 | 11.83 |
|  |  | High | 15.66 | 15.66 | 15.66 | 15.66 | 16.70 | 17.75 | 17.75 | 17.75 |
|  | Profit (in million 2018 US$) | Low | 197.64 | 197.64 | 197.64 | 197.64 | 210.81 | 223.99 | 223.99 | 223.99 |
|  |  | High | 305.70 | 305.70 | 305.70 | 305.70 | 326.08 | 346.46 | 346.46 | 346.46 |
| **GAVI** | Price | | 4.69 | 4.69 | 4.69 | 4.69 | 4.69 | 4.69 | 4.69 | 4.69 |
|  | Variable costs | Low | 1.00 | 1.00 | 1.00 | 1.00 | 1.00 | 1.00 | 1.00 | 1.00 |
|  |  | High | 1.59 | 1.59 | 1.59 | 1.59 | 1.59 | 1.59 | 1.59 | 1.59 |
|  | # doses (in millions) | Low | 27.84 | 21.58 | 22.97 | 25.06 | 17.40 | 22.27 | 20.88 | 20.18 |
|  |  | High | 41.76 | 32.36 | 34.45 | 37.58 | 26.10 | 33.41 | 31.32 | 30.28 |
|  | Profit (in million US$) | Low | 86.23 | 66.83 | 71.14 | 77.61 | 53.90 | 68.99 | 64.68 | 62.52 |
|  |  | High | 153.99 | 119.34 | 127.04 | 138.59 | 96.24 | 123.19 | 115.49 | 111.64 |
| **Indonesia and India** | Price | | 11.28 | 11.28 | 11.28 | 11.28 | 11.28 | 11.28 | 11.28 | 11.28 |
|  | Variable costs | Low | 1.00 | 1.00 | 1.00 | 1.00 | 1.00 | 1.00 | 1.00 | 1.00 |
|  |  | High | 1.59 | 1.59 | 1.59 | 1.59 | 1.59 | 1.59 | 1.59 | 1.59 |
|  | # doses (in millions) | Low | 0.70 | 11.14 | 20.18 | 18.79 | 19.49 | 17.40 | 16.01 | 14.62 |
|  |  | High | 1.04 | 16.70 | 30.28 | 28.19 | 29.23 | 26.10 | 24.01 | 21.92 |
|  | Profit (in million 2018 US$) | Low | 6.75 | 107.92 | 195.61 | 182.12 | 188.86 | 168.63 | 155.14 | 141.65 |
|  |  | High | 10.73 | 171.74 | 311.28 | 289.81 | 300.54 | 268.34 | 246.87 | 225.41 |
| **China** | Price | |  |  | 159.38 | 159.38 | 159.38 | 159.38 | 159.38 | 159.38 |
|  | Variable costs | Low |  |  | 1.00 | 1.00 | 1.00 | 1.00 | 1.00 | 1.00 |
|  |  | High |  |  | 1.59 | 1.59 | 1.59 | 1.59 | 1.59 | 1.59 |
|  | # doses (in millions) | Low | 0.00 | 0.00 | 0.70 | 2.09 | 2.78 | 2.78 | 3.48 | 4.87 |
|  |  | High | 0.00 | 0.00 | 1.04 | 3.13 | 4.18 | 4.18 | 5.22 | 7.31 |
|  | Profit (in million 2018 US$) | Low | 0.00 | 0.00 | 109.82 | 329.46 | 439.27 | 439.27 | 549.09 | 768.73 |
|  |  | High | 0.00 | 0.00 | 165.34 | 496.03 | 661.37 | 661.37 | 826.72 | 1,157.40 |
| **Total profit (in million 2018 US$)** | | **Low** | **2,201.99** | **2,183.17** | **2,284.38** | **2,396.40** | **2,401.83** | **2,309.26** | **2,401.28** | **2,605.27** |
|  |  | **High** | **3,349.18** | **3,324.03** | **3,485.09** | **3,654.35** | **3,656.94** | **3,520.56** | **3,656.73** | **3,962.10** |

*Source*: Own calculations based on Tables S1, S2, S5a, S5b and Table 3.

We next present the details for the estimation of fixed manufacturing costs that are displayed in Table 5. Table S10a starts with the derivation of annual fixed labor compensation, based on hourly compensation and the required number of directors, managers and supervisors per factory as provided by Clendinen *et al.* [12]. Factory and administrative overhead (in 2018 US$) costs are computed in Table S10b, assumed to be 45% of the sum of total labor costs plus the costs for materials. From 2022 onwards, the total fixed manufacturing cost is based on three factories rather than one. The assumptions are discussed in Section 2.3 (main text).

**Table S10a.** Annual manufacturing fixed costs, in 2014 US$ – detailed derivation of Table 5.

|  | **Personnel** | **Annual Compensation** | | **Total Costs** | |
| --- | --- | --- | --- | --- | --- |
| **Fixed number of employees per factory** | **Type** | **Low** | **High** | **Low** | **High** |
| 1 | Director | 150,000 | 200,000 | 150,000 | 200,000 |
| 3 | Managers | 100,000 | 150,000 | 300,000 | 450,000 |
| 7 | Supervisors | 70,000 | 100,000 | 490,000 | 700,000 |
| Fixed costs for indicated personnel (in 2014 US$) | | | | 940,000.00 | 1,350,000.00 |
| **Fixed costs for indicated personnel (in 2018 US$)**** | | | | **1,000,000.00** | **1,436,170.21** |
| **Factory and administrative overhead (in 2018 US$)*** | | | | **7,740,365.58** | **11,962,632.65** |
| **Total fixed costs per factory per year (in 2018 US$) (2007-2021)** | | | | **8,740,365.58** | **13,398,802.86** |
| **Total fixed costs in 3 factories per year (from 2022)** | | | | **26,221,096.74** | **40,196,408.58** |

* Derived in Table S10b. ** Deflated by price index for 2014, PI=0.942 (Table S0).

*Source:* Clendinen *et al.* [12] and own calculations.

**Table S10b.** Low and high estimate for the factory and administrative overhead, in 2018 US$.

|  | | **Low estimate** | **High estimate** |
| --- | --- | --- | --- |
| **Annual personnel cost** | **Fixed*** | 1,000,000 | 1,436,170 |
|  | **Variable (cost of 1 million x 28.55)**** | 6,952,110 | 9,732,955 |
| **Cost of materials (cost of 1 million x 28.55)**** | | 9,248,702 | 15,414,503 |
| **Total** | | 17,200,812 | 26,583,628 |
| **Annual factory and administrative overhead costs (45% costs of personnel and material)** | | 7,740,366 | 11,962,633 |

* Manufacturing fixed costs are taken from Table S10a. ** For variable costs of labor and materials per one million doses, see Table 3. These are multiplied by the average number of doses sold per year between 2010-2017 (i.e. 228.38/8=28.55 million, according to Table S3b).

*Source:* Clendinen *et al.* [12] and own calculations.

The information from Table S10a is taken over in the left panel of Table S11 to summarize manufacturing fixed costs. The right panel of Table S11 presents the sum of DTCA spending in the U.S (90% of global DTCA spending) and the estimates for marketing expenditure on health providers (150% and 50% of global DTCA spending for the “high estimate” and “low” estimate, respectively); see Section 2.3. The information in Table S11 is used by applying equation No. 3 to compute the PDV of the stream of fixed manufacturing and marketing costs from 2020-2028 and 2007-2028. Results are presented in Table 6 of the main text.

**Table S11.** Estimated fixed manufacturing and marketing costs from 2007-2028, in million 2018 US$.

|  | **Manufacturing fixed costs*** | | **Marketing costs**** | |
| --- | --- | --- | --- | --- |
|  | **High estimate** | **Low estimate** | **High estimate** | **Low estimate** |
| **2007** | 13.399 | 8.740 | 502.81** | 302.05** |
| **2008** | 13.399 | 8.740 | 334.05 | 200.67 |
| **2009** | 13.399 | 8.740 | 218.30 | 131.14 |
| **2010** | 13.399 | 8.740 | 168.83 | 101.42 |
| **2011** | 13.399 | 8.740 | 134.58 | 80.85 |
| **2012** | 13.399 | 8.740 | 137.81 | 82.79 |
| **2013** | 13.399 | 8.740 | 152.27 | 91.47 |
| **2014** | 13.399 | 8.740 | 211.06 | 126.79 |
| **2015** | 13.399 | 8.740 | 213.92 | 128.51 |
| **2016** | 13.399 | 8.740 | 140.27 | 84.27 |
| **2017** | 13.399 | 8.740 | 8.89 | 5.34 |
| **2018** | 13.399 | 8.740 | 36.02 | 21.64 |
| **2019** | 13.399 | 8.740 | 1.20 | 0.72 |
| **2020** | 13.399 | 8.740 | 15.37 | 9.23 |
| **2021** | 13.399 | 8.740 | 15.37 | 9.23 |
| **2022-2028** | 40.196 | 26.221 | 15.37 | 9.23 |

* From Table S10a. ** We assume that marketing spending on health providers is 50% (“low” scenario) and 150% (“high” scenario) of the direct to consumer advertising (DTCA) spending, respectively, and that 90% of the marketing spending is in the U.S. Moreover, marketing costs in 2007 (the first full year Gardasil was sold) include costs of 2006 and the annual marketing costs for the period 2020-2028 equals the average of the annual marketing costs from 2017-2019. DTCA spending originally in current Euro. All numbers converted to current US$ using the exchange rate from Thomson Reuters and then deflated by PI from Table S0.

*Source:* Clendinen *et al.* [12], ‘The Nielsen Company’ (DTCA spending for the U.S.), and own calculations.

We calculate R&D costs for each phase as given in Table 8 as follows. We use the bounds of the cost range per subject provided by Light *et al.* [15] as given in Table S12 and multiply them by the number of subjects as given in Table S14a for Gardasil-4 and S14b for Gardasil-9. Analogously, we use the site cost and study cost estimates by Sertkaya *et al.* [21] given in Table S13 jointly with Tables S14a and S14b. As Sertkaya *et al.* [21] originally provide average costs from 2004-2012 in current US$, we use the price index PI in Table S0 for the median year 2008 to calculate the corresponding costs in 2018 US$.

**Table S12. Cost per subject per clinical trial phase**

|  | **Cost per subject**  **(in 2008 US$)** | | **Cost per subject**  **(in 2018 US$)*** | |
| --- | --- | --- | --- | --- |
|  | **Low** | **High** | **Low** | **High** |
| **Phase I** | 100.00 | 400.00 | 116.28 | 465.12 |
| **Phase II** | 300.00 | 400.00 | 348.84 | 465.12 |
| **Phase III** | 2,000.00 | 3,000.00 | 2,325.58 | 3,488.37 |

* Deflated by PI=0.86 for year 2008 (Table S0).

*Source:* Light *et al.* [15].

**Table S13.** Site and study costs estimates for each phase of the clinical trial.

|  | **Phase I** | **Phase II** | **Phase III** |
| --- | --- | --- | --- |
| **Site costs** | | | |
| Recruitment | 51,904.00 | 233,729.00 | 395,182.00 |
| Site retention | 193,615.00 | 1,127,005.00 | 1,305,361.00 |
| Administrative staff | 237,869.00 | 1,347,390.00 | 2,321,628.00 |
| Monitoring | 198,896.00 | 1,083,186.00 | 1,624,874.00 |
| **Total (in 2008 US$)** | **682,284.00** | **3,791,310.00** | **5,647,045.00** |
| **Total (in 2018 US$)** | **793,353.49** | **4,408,500.00** | **6,566,331.40** |
| **Study costs** | | | |
| Data management | 50,331.00 | 59,934.00 | 39,047.00 |
| IRB approvals | 11,962.00 | 60,188.00 | 114,118.00 |
| IRB amendments | 1,094.00 | 1,698.00 | 1,919.00 |
| Source data verification | 326,437.00 | 406,038.00 | 400,173.00 |
| Overheads | 528,685.00 | 1,741,811.00 | 2,541,313.00 |
| Other costs | 1,139,887.00 | 4,003,615.00 | 5,967,193.00 |
| **Total (in 2008 US$)** | **2,058,396.00** | **6,273,284.00** | **9,063,763.00** |
| **Total (in 2018 US$)*** | **2,393,483.72** | **7,294,516.28** | **10,539,259.30** |

* Deflated by PI=0.86 for year 2008 (Table S0).

*Source*: Sertkaya *et al.* [21].

**Table S14a.** Estimated costs of R&D from phase I to phase III clinical trial for Gardasil-4, in 2018 US$.

| **Phase** | # | **Description** | **# of subjs** | **Costs estimation per subject*** | | **Calculated costs** | |
| --- | --- | --- | --- | --- | --- | --- | --- |
|  |  |  |  | Low | High | Low | High |
| **Phase I** | 1*** | • In one study subjects were given four dose formulations of HPV11 L1 VLP vaccine (10, 20, 50, and 100 ug). There were 28 subjects per dose level and 28 for the placebo group • In the other study three different formulations were used to test HPV16 L1 VLPs: 10 ug (13 active and four placebo), 40 ug (45 active and 15 placebo) and 80 ug (24 active and eight placebo).  • Source: [88] | 249 | 116 | 465 | 28,953 | 115,814 |
|  | 2*** | • 40 women, aged 16-23 years, were randomly assigned (2:1 vaccine to placebo ratio) to receive either HPV18 L1 VLP vaccine or placebo.  •Source: [89] | 40 | 116 | 465 | 4,651 | 18,605 |
|  | 3*** | • Healthy nonpregnant women aged 18 to 26 years old were assigned to study groups to receive placebo or a 3-dose regime of the different HPV 16 L1 VLP vaccine dosage of 10 μg (n=112), 20 μg (n=105), 40 μg (n=104), or 80 μg (n=107).  • Source: [90] | 480 | 116 | 465 | 55,814 | 223,256 |
|  | 4 | • Females aged 9-26 year were vaccinated with a single dose of Gardasil in an open label study to evaluate safety and tolerability of the vaccine. • From: March 2008-April 2008 • ID: NCT00635830 | 40 | 116 | 465 | 4,651 | 18,605 |
|  | **Total spent on subjects on phase I** | | | | | 94,070 | 376,279 |
|  | **Total spent on sites (number of studies x 793,353.49**)** | | | | | 3,173,414 | 3,173,414 |
|  | **Total spent of study costs (number of studies x 2,393,483.72**)** | | | | | 9,573,935 | 9,573,935 |
|  | **Total spent on phase I clinical trials** | | | | | 12,841,419 | 13,123,628 |
| **Phase II** | 1 | • Young women aged 16-23 years old were randomly assigned to receive three doses of placebo (n=1198) or HPV-16 virus-like–particle vaccine (n=1194) • From: September 1999- March 2004 • ID: NCT00365378 | 2,392 | 349 | 465 | 834,419 | 1,112,558 |
|  | 2 | • A total of 831 women aged 16-23 years were vaccinated with one of the three formulations quadrivalent HPV (Types 6/11/16/18) L1 virus-like particle (VLP) (each of the 3 groups had 275-280 subjects) or received one of the two placebo formulations (n=275). Dose escalation assessment (n=52). • From: May 2000-May 2004  • ID: NCT00365716 | 1,158 | 349 | 465 | 403,953 | 538,605 |
|  | 3 | • Women aged 18-26 years were assigned to receive Gardasil vaccination (n=509) or placebo (n=512) • From: June 2006-September 2009 • ID: NCT00378560 | 1,021 | 349 | 465 | 356,163 | 474,884 |
|  | 4 | • Evaluate the effectiveness of Gardasil in men aged 27-45 who have completed 4 years of observation in HPV infection in men • Duration: December 2012 - October 2019 • ID: NCT01432574 | 150 | 349 | 465 | 52,326 | 69,767 |
|  | 5 | • The immunogenicity, safety and tolerability of the quadrivalent vaccine was assessed in females Aged 9-17 years. • From: December 2006 - September 2009 • ID: NCT00411749 | 107 | 349 | 465 | 37,326 | 49,767 |
|  | **Total spent on subjects on phase II** | | | | | **1,684,186** | **2,245,581** |
|  | **Total spent on sites (number of studies x 4,408,500.00**)** | | | | | 22,042,500 | 22,042,500 |
|  | **Total spent of study costs (number of studies x 7,294,516.28**)** | | | | | 36,472,581 | 36,472,581 |
|  | **Total spent on phase II clinical trials** | | | | | **60,199,267** | **60,760,663** |
| **Phase III** | 1 | • Women aged 16-24 year were randomly assigned to receive 3 doses of the quadrivalent vaccine (2723) or placebo (n=2732).  • From: December 2001-January 2009; ID: NCT00092521 | 5,455 | 2,326 | 3,488 | 12,686,047 | 19,029,070 |
|  | 2 | • Women aged 16-23 were randomized (1:1:1:1) to receive three doses of quadrivalent HPV-6/11/16/18 vaccine co-administered with HBV vaccine, quadrivalent vaccine with HBV-vaccine matched placebo, HBV vaccine with HPV-vaccine matched placebo, or HPV-vaccine matched placebo and HBV-vaccine matched placebo. • From: December 2001 - June 2004 • ID: NCT00517309 | 1,871 | 2,326 | 3,488 | 4,351,163 | 6,526,744 |
|  | 3 | • Women aged 16-23 years in a phase III study to compare the immunogenicity and safety of the quadrivalent Gardasil and Monovalent HPV 16 vaccine. • From: June 2002 - June 2004 • ID: NCT00092482 | 3,882 | 2,326 | 3,488 | 9,027,907 | 13,541,860 |
|  | 4 | • Women aged 15-26 were randomly assigned 1:1 to receive 3 doses of the quadrivalent vaccine or placebo • From: June 2002 - July 2007 • ID: NCT00092534 | 12,167 | 2,326 | 3,488 | 28,295,349 | 42,443,023 |
|  | 5 | • Women age 10-23 years were randomly assigned to receive placebo or Gardasil to assess the immune response to the 4 components of the vaccine. • From: December 2002 - September 2004 • ID: NCT00092495 | 3,055 | 2,326 | 3,488 | 7,104,651 | 10,656,977 |
|  | 6 | • Adolescents aged 9 to 15 years were randomly assigned 2:1 to receive HPV4 vaccine or saline placebo. On the 30th, the placebo group (n = 482) received the same regimen of HPV4 vaccine and both cohorts were followed through month 96.  • From: October 2003 - November 2005 • ID: NCT00092547 | 1,781 | 2,326 | 3,488 | 4,141,860 | 6,212,791 |
|  | 7 | • Women aged 24-45 years were receive 3 doses of Gardasil (n=1911) or placebo (1908). • ID: NCT00090220 • From: June 2004 - May 2009 | 3,819 | 2,326 | 3,488 | 8,881,395 | 13,322,093 |
|  | 8 | • Heterosexual males aged 16-24 (n=3463) and homosexual men aged 16–24 years (n= 602) were randomly assigned to receive three doses of Gardasil (n=2032) or placebo (n=2033). • From: September 2004 - July 2009 • ID: NCT00090285 | 4,065 | 2,326 | 3,488 | 9,453,488 | 14,180,233 |
|  | 9 | • Females aged 9–23 years were randomly assigned to receive three doses of Gardasil (n=117) or placebo (n=59). • From: October 2005 - June 2006 • ID: NCT00157950 | 176 | 2,326 | 3,488 | 409,302 | 613,953 |
|  | 10 | • Adolescents (394 boys and 648 girls) aged 10-17 years were randomly assigned in a 1:1 ratio to receive: 3 doses of Gardasil with one dose of Menactra and Adacel (concomitant), 3 of Gardasil with one dose of Menactra and Adacel (nonconcomitant). • From: April 2006 - April 2007 • ID: NCT00325130 | 1,042 | 2,326 | 3,488 | 2,423,256 | 3,634,884 |
|  | 11 | • Teenage boys and girls aged 11-17 were enrolled in an open-label study in which all subjects received three doses of GARDASIL and one of REPEVAX. • From: May 2006-May 2007 • ID: NCT00337428 | 843 | 2,326 | 3,488 | 1,960,465 | 2,940,698 |
|  | 12 | • Women aged 9-15 years participated in the study to evaluate the safety and tolerability of Gardasil. • From: May 2007-February 2008 • ID: NCT00380367 | 110 | 2,326 | 3,488 | 255,814 | 383,721 |
|  | 13 | • Chinese females aged 9-45 years (n=500) and males aged 9 to 15 years (n=100) were randomly assigned in a 1:1 ratio to receive either 3 doses of Gardasil or aluminum-containing placebo.  • From: July 20, 2008-February 28, 2009 • ID: NCT00496626 | 600 | 2,326 | 3,488 | 1,395,349 | 2,093,023 |
|  | 14 | • Females aged 20-45 years were assigned to receive three doses of Gardasil or placebo to test the safety and effectiveness of the vaccine. • From: December 31, 2008- May 11, 2012 • ID: NCT00834106 | 3,006 | 2,326 | 3,488 | 6,990,698 | 10,486,047 |
|  | 15 | • Sub-Saharan females aged 9-26 were enrolled in the study to evaluate safety, tolerability and immunogenicity of the three dose Gardasil. Thirty females ages 13-15 and 120 females ages 16-26 received the three dose Gardasil. In addition, girls aged 9-12 years were randomized in a 4:1 ratio to receive either Gardasil (n = 80) or placebo (n = 20). • From: March 2011 - April 2013 • ID: NCT01245764 | 250 | 2,326 | 3,488 | 581,395 | 872,093 |
|  | 16 | • Open label study to evaluate Gardasil`s safety and effectiveness in females aged 16- to 26 years. • From: November 2011-August 2016 • ID: NCT01544478 | 1,030 | 2,326 | 3,488 | 2,395,349 | 3,593,023 |
|  | 17 | • Japanese males aged 16-26 year were enrolled in a study to evaluate the efficacy and tolerability of Gardasil. • From: June 2013 - August 2017 • ID: NCT01862874 | 1,124 | 2,326 | 3,488 | 2,613,953 | 3,920,930 |
|  | 18 | • Evaluate the immunogenicity, safety, and tolerability of Gardasil in females aged 9-26 years  • Duration: August 2018 - October 2023 • ID: NCT03493542 | 766 | 2,326 | 3,488 | 1,781,395 | 2,672,093 |
|  | 19 | • Evaluate Two-dose schedule of Gardasil-4 in 11-year-old Boys • Duration: February 2015 - December 2015 • ID: NCT02382900 | 500 | 2,326 | 3,488 | 1,162,791 | 1,744,186 |
|  | 20 | • Evaluate tolerability and immunogenicity of a 3-dose regimen of Gardasil administered to healthy married females aged 16-23 years  • Duration: October 2009 - October 2013 • ID: NCT00733122 | 600 | 2,326 | 3,488 | 1,395,349 | 2,093,023 |
|  | 21 | • Boys aged 9-15 years were enrolled in an open label two-part study in which part 1 assessed immunogenicity and tolerability of Gardasil up to Month 7 whereas part 2 assessed long-term immunogenicity and safety (Month 7-Month 30).  • From: November 2015 - August 2018 • ID: NCT02576054 | 100 | 2,326 | 3,488 | 232,558 | 348,837 |
|  | **Total spent on subjects on phase III** | | | | | **107,539,535** | **161,309,302** |
|  | **Total spent on sites (number of studies x 6,566,331.40**)** | | | | | 137,892,959 | 137,892,959 |
|  | **Total spent of study costs (number of studies x 10,539,259.30**)** | | | | | 221,324,445 | 221,324,445 |
|  | **Total spent on phase III clinical trials** | | | | | **466,756,940** | **520,526,707** |
|  | **Total cost of all phases** | | | | | **539,797,626** | **594,410,998** |

* From estimates displayed in Table S12. ** From estimates displayed in Table S13. *** Clinical trial was not registered on [www.clinicaltrial.gov](http://www.clinicaltrial.gov) thus they do not have an ID. However, results were published in peer reviewed journals cited in the description (“Source”).

**Table S14b.** Estimated costs of R&D from phase I to phase III clinical trial for Gardasil-9, in 2018 US$.

| **Phase** | # | **Description** | **# of subjs** | **Costs estimation per subject*** | | **Calculated costs** | |
| --- | --- | --- | --- | --- | --- | --- | --- |
|  |  |  |  | Low | High | Low | High |
| **Phase I** | 1 | • Evaluate the safety and tolerability of octavalent HPV L1 VLP vaccine formulated with amorphous aluminum hydroxysulfate and ISCOMATRIX in females aged 18-24 years • Duration: April 2006 - November 2009 • ID: NCT00851643 | 158 | 116 | 465 | 18,372 | 73,488 |
|  | **Total spent on subjects on phase I** | | | | | 18,372 | 73,488 |
|  | **Total spent on sites (number of studies x 793,353.49**)** | | | | | 793,353 | 793,353 |
|  | **Total spent of study costs (number of studies x 2,393,483.72**)** | | | | | 2,393,484 | 2,393,484 |
|  | **Total spent on phase I clinical trials** | | | | | **3,202,637** | **3,250,037** |
| **Phase II** | 1 | • Determine immunogenicity, safety and tolerability of Gardasil-4 and 9 vaccine in young cancer survivors aged 9-26 years • Duration: July 2012 - November 2020 • ID: NCT01492582 | 1252 | 349 | 465 | 436,744 | 582,326 |
|  | 2 | • Females aged 16-23 years were enrolled in a study to evaluate the tolerability and immunogenicity of the 3-dose vaccine. • From: December 2005 - August 2007 • ID: NCT00260039 | 680 | 349 | 465 | 237,209 | 316,279 |
|  | 3 | • Compare safety and immunogenicity of V505 HPV vaccine candidate and Gardasil-4 in females 16-26 years • Duration: October 2007-May 2011 • ID: NCT00520598 | 511 | 349 | 465 | 178,256 | 237,674 |
|  | 4 | • Examine tolerability and immunogenicity HPV L1 VLP vaccine candidate administered Concomitantly with Gardasil in females aged 16-26. • Duration: October 2007 - May 2009 • ID: NCT00551187 | 620 | 349 | 465 | 216,279 | 288,372 |
|  | **Total spent on subjects on phase II** | | | | | 1,068,488 | 1,424,651 |
|  | **Total spent on sites (number of studies x 4,408,500.00*)** | | | | | 17,634,000 | 17,634,000 |
|  | **Total spent of study costs (number of studies x 7,294,516.28*)** | | | | | 29,178,065 | 29,178,065 |
|  | **Total spent on phase II clinical trials** | | | | | 47,880,553 | 48,236,716 |
| **Phase III** | 1 | • A Phase III Open-label Safety and Immunogenicity Study of GARDASIL™9 Administered to 9- to 26-Year-Old Females and Males in Vietnam • Duration: June 2018 - January 2019 • ID: NCT03546842 | 200 | 2,326 | 3,488 | 465,116 | 697,674 |
|  | 2 | • This study will assess the safety and immunogenicity of GARDASIL®9 (V503) in 27- to 45-year-old women  • Duration: September 2017 - November 2018 • ID: NCT03158220 | 1212 | 2,326 | 3,488 | 2,818,605 | 4,227,907 |
|  | 3 | • Examine the acceptability, uptake and immunogenicity of the vaccine in the postpartum setting in women 16 years to 26 years • Duration: November 2018 - July 2019 • ID: NCT03451071 | 200 | 2,326 | 3,488 | 465,116 | 697,674 |
|  | 4 | • Assess occupational exposure to Human Papilloma Virus (HPV) and prophylactic vaccination in healthcare workers aged 27-69 • Country: USA • Duration: February 2018 - November 2018 • ID: NCT03350698 | 100 | 2,326 | 3,488 | 232,558 | 348,837 |
|  | 5 | • Evaluate the Immunogenicity of the nonvalent vaccine against Human Papillomavirus in men (age 18-36 years) infected by HIV who have sex with men. • Duration: October 2018 - December 2021 • ID: NCT03626467 | 166 | 2,326 | 3,488 | 386,047 | 579,070 |
|  | 6 | • Assess the efficacy of HPV vaccine in reducing high-grade cervical lesions in patients with HPV and HIV infections in females aged 25 and older • Duration: January 2019 - October 2021 • ID: NCT03284866 | 536 | 2,326 | 3,488 | 1,246,512 | 1,869,767 |
|  | 7 | • Assess the safety and immunogenicity of a 2-dose regimen of Gardasil-9 (V503) in boys and girls 9 to 14 years of age and in young women aged 16-26 years  • Duration: November 2013 - August 2018 • ID: NCT01984697 | 1518 | 2,326 | 3,488 | 3,530,233 | 5,295,349 |
|  | 8 | • Assess safety, immunogenicity and long-term effectiveness Gardasil-9 in preventing cervical cancer and related precancers caused by HPV types covered in the vaccine in females aged 16-26 years  • Duration: January 2016 - January 2024 • ID: NCT02653118 | 4453 | 2,326 | 3,488 | 10,355,814 | 15,533,721 |
|  | 9 | • Evaluate immunogenicity and tolerability of Gardasil-9 administered Concomitantly with Menactra and Adacel in boys and girls aged 11-15 year • Duration: October 2009 - February 2011 • ID: NCT00988884 | 1241 | 2,326 | 3,488 | 2,886,047 | 4,329,070 |
|  | 10 | • Evaluate tolerability of Gardasil-9 in females aged 12-26 years who were previously vaccinated with GARDASIL-4 • Duration: February 2010 - November 2015 • ID: NCT01047345 | 924 | 2,326 | 3,488 | 2,148,837 | 3,223,256 |
|  | 11 | • Evaluate if Gardasil-9 induces non-inferior Geometric Mean Titres (GMTs) for serum anti-HPV 6, 11, 16, and 18, compared to GARDASIL-4 in males aged 16 – 26 year • Duration: March 2014 - April 2015 • ID: NCT02114385 | 500 | 2,326 | 3,488 | 1,162,791 | 1,744,186 |
|  | 12 | • Compare immunogenicity and tolerability of Gardasil-4 and 9 in females ages 9-15 years • Duration: February 2011 - December 2011 • ID: NCT01304498 | 600 | 2,326 | 3,488 | 1,395,349 | 2,093,023 |
|  | 13 | • Evaluate whether if first dose of Gardasil-9 concomitantly administrated with REPEVAX™ is well tolerated and equally immunogenic compared to administration of REPEVAX a month after Gardasil-9 first dose • Countries: Finland, Germany, Denmark, Thailand, Belgium, Austria • Duration: April 2010 - June 2011 • ID: NCT01073293 | 1054 | 2,326 | 3,488 | 2,451,163 | 3,676,744 |
|  | 14 | • Evaluate safety, tolerability and Immunogenicity of Gardasil-9 in Japanese girls aged 9-15 year • Duration: January 2011 - August 2013 • ID: NCT01254643 | 100 | 2,326 | 3,488 | 232,558 | 348,837 |
|  | 15 | • Assess immunogenicity and tolerability of Gardasil-9 in males and females aged 9-15 years • Duration: August 2009 - December 2020 • ID: NCT00943722 | 3074 | 2,326 | 3,488 | 7,148,837 | 10,723,256 |
|  | 16 | • Compare the safety, efficacy, and immunogenicity of Gardasil-4 and 9 in females aged 16-26 years old. • Duration: September 2007 - July 2016 • ID: NCT00543543 | 14840 | 2,326 | 3,488 | 34,511,628 | 51,767,442 |
|  | 17 | • Evaluate immunogenicity and tolerability of Gardasil-9 in males and females aged 16-26 years • Duration: October 2012 - August 2014 • ID: NCT01651949 | 2520 | 2,326 | 3,488 | 5,860,465 | 8,790,698 |
|  | **Total spent on subjects on phase III** | | | | | **77,297,674** | **115,946,512** |
|  | **Total spent on sites (number of studies x 6,566,331.40**)** | | | | | 111,627,634 | 111,627,634 |
|  | **Total spent of study costs (number of studies x 10,539,259.30**)** | | | | | 179,167,408 | 179,167,408 |
|  | **Total spent on phase III clinical trials** | | | | | **368,092,716** | **406,741,553** |
|  | **Total costs of all phases** | | | | | **419,178,479** | **458,238,595** |

* From estimates displayed in Table S12. ** From estimates displayed in Table S13.

**References – Supporting Information**

88. Fife KH, Wheeler CM, Koutsky LA, Barr E, Brown DR, Schiff MA, et al. Dose-ranging studies of the safety and immunogenicity of human papillomavirus Type 11 and Type 16 virus-like particle candidate vaccines in young healthy women. Vaccine [Internet]. 2004 Jul 29 [cited 2018 Oct 29];22(21–22):2943–52. Available from: https://www.sciencedirect.com/science/article/pii/S0264410X04000179#aep-acknowledgment-id34

89. Ault KA, Giuliano AR, Edwards RP, Tamms G, Kim L-L, Smith JF, et al. A phase I study to evaluate a human papillomavirus (HPV) type 18 L1 VLP vaccine. Vaccine [Internet]. 2004 Aug 13 [cited 2018 Oct 29];22(23–24):3004–7. Available from: https://www.sciencedirect.com/science/article/pii/S0264410X04001823

90. Poland GA, Jacobson RM, Koutsky LA, Tamms GM, Railkar R, Smith JF, et al. Immunogenicity and Reactogenicity of a Novel Vaccine for Human Papillomavirus 16: A 2-Year Randomized Controlled Clinical Trial. Mayo Clin Proc [Internet]. 2005 May 1 [cited 2018 Oct 29];80(5):601–10. Available from: https://www.sciencedirect.com/science/article/abs/pii/S0025619611630917
